# CD4^+^FoxP3^+^CD73^+^ regulatory T cell promotes cardiac healing post-myocardial infarction

**DOI:** 10.7150/thno.68437

**Published:** 2022-03-06

**Authors:** Rulin Zhuang, Qingshu Meng, Xiaoxue Ma, Shanshan Shi, Shiyu Gong, Jing Liu, Mimi Li, Wenchao Gu, Dan Li, Xumin Zhang, Zhulin Wang, Xinyu Ge, Jiayou Tang, Fang Lin, Xiaoting Liang, Liang Zheng, Zhongmin Liu, Xiaohui Zhou

**Affiliations:** 1 Department of Cardiovascular Surgery, Shanghai East Hospital, Tongji University School of Medicine, Shanghai, 200120, China; 2 Research Center for Translational Medicine, Shanghai East Hospital, Tongji University School of Medicine, Shanghai, 200120, China; 3 Institute of Integrated Traditional Chinese and Western Medicine for Cardiovascular Chronic Diseases, Tongji University School of Medicine, Shanghai, 200120, China; 4 Shanghai Heart Failure Research Center, Shanghai, 200120, China; 5 Department of Cardiology, Shanghai East Hospital, Tongji University School of Medicine, Shanghai, 200120, China; 6 Department of Immunology and Microbiology, Shanghai Institute of Immunology, Shanghai Jiao Tong University School of Medicine, Shanghai, China; 7 Department of Child Internal Medicine, Shanghai Children's Medical Center, Shanghai Jiaotong University, Shanghai 200127, China; 8 Translational Medical Center for Stem Cell Therapy, Shanghai East Hospital, Tongji University, Shanghai 200120, China; 9 Shanghai Institute of Stem Cell Research and Clinical Translation, Shanghai 200120, China

**Keywords:** Regulatory T cells (Tregs), ecto-5'-nucleotidase (CD73), myocardial infarction (MI), cardiac healing, IL-2/anti-IL-2 complex

## Abstract

**Rationale:** Despite recent studies indicating a crucial role of ecto-5′-nucleotidase (CD73) on T cells in cardiac injury after ischemia/reperfusion, the involvement of CD73^+^ regulatory T cells (Tregs) in cardiac repair post-myocardial infarction (MI) remains unclear. We sought to investigate the contribution of CD73 on Tregs to the resolution of cardiac inflammation and remodeling after MI.

**Methods:** Cardiac function, tissue injury, Tregs percentage in injured hearts, and purinergic signaling changes in cardiac FoxP3^+^ Tregs were analyzed after permanent descending coronary artery ligation. CD73 knockout Tregs were used to determine the function of CD73 on Tregs. Peripheral blood mononuclear cells (PBMCs) from acute myocardial infarction (AMI) patients and matched non-MI subjects were assessed via flow cytometry.

**Results:** Cardiac Tregs exhibited distinction of purinergic signaling post MI with dramatically high level of CD73 compared to the sham Tregs. CD73 deficiency decreased the tissue tropism, and impaired the immunosuppressive and protective function of Tregs in cardiac healing. Administration of low-dose of IL-2/anti-IL-2 complex resulted in FoxP3^+^CD73^+^Tregs expansion in the heart and contributed to the recovery of cardiac function. CD73 derived from FoxP3^+^Tregs could bind to FoxP3^-^ effector T-cells and inhibit the production of multiple inflammatory cytokines. In AMI patients, CD73 expressions on both CD4^+^ cells and FoxP3^+^Tregs decreased in PBMCs. Moreover, CD73 expressions on CD4^+^ T cells were negatively correlated with the levels of NT pro-BNP and myocardial zymogram in serum.

**Conclusions:** Our findings indicated the importance of FoxP3^+^CD73^+^Tregs in inflammation resolution and cardiac healing post-MI.

## Introduction

Excessive and persistent inflammation after myocardial infarction (MI) triggers subsequent left ventricular (LV) remodeling and heart failure (HF). In the past decade, the importance of T cell-mediated chronic inflammation has been highlighted in the progression of various cardiovascular diseases including hypertension, atherosclerosis, MI, and HF 1, 2. Infiltration of T cells in the LV has been shown to result in nonischemic HF progression in both patients and murine models 3. Also, the critical role of CD4^+^ T cell was confirmed in heart regeneration and repair during development recently 4. However, the role of T cell in cardiac ischemic injury still remains controversial. One study reported that activated T cell is an essential driver of pathological remodeling 5, while another suggested that activation of CD4^+^ T cell is a prerequisite for proper wound healing and subsequent remodeling of the myocardium in murine MI models 6. Regulatory T cells (Tregs), a specialized subset of T cell, can mediate immune homeostasis by blocking excessive immune responses, inflammation, and tissue destruction 7, 8. The emerging role of Tregs was also confirmed in the heart regeneration by enhancing cardiomyocyte proliferation recently 9, 10. In the murine MI model, Tregs are typically recruited to the heart and exert a beneficial role in cardiac remodeling early after injury 11. Depletion of Tregs led to exacerbated cardiac inflammation and dysfunction, whereas expansion of this subtype via super-agonistic anti-CD28 monoclonal antibody 12, interleukin-2 and anti-interleukin-2 antibody complex (IL2C) 13, or adoptive transfer of Tregs 14, 15 contributed to heart healing post-MI 16. Collectively, these studies support the notion that Tregs could be a promising therapeutic target for post-MI cardiac repair.

Accumulating studies revealed the vital role of purinergic signaling in T cell functions. Adenosine triphosphate (ATP), released by dying and damaged cells, acts as an immunostimulatory signal that promotes inflammatory responses. Ectonucleoside triphosphate diphosphohydrolase-1 (CD39) and ecto-5′-nucleotidase (CD73) can degrade ATP to adenosine diphosphate (ADP), adenosine monophosphate (AMP), and eventually to the anti-inflammatory mediator adenosine, which further inhibits the activation, differentiation, and cytokine production of T cell 17, 18. The expression of CD73 and CD39 on Tregs is necessary for the immunosuppressive function of Tregs in allograft rejection [19]and inflammatory autoimmune diseases 20. Moreover, global deficiency of CD73 worsened cardiac dysfunction after ischemia/reperfusion (I/R), and was accompanied by a prolonged inflammatory response and enhanced fibrosis 21. In murine HF models induced by transverse aortic constriction (TAC), lack of CD73 on T cells deteriorated the heart contractile functions and increased cardiac fibrosis when compared to wild-type (WT) mice 22. Recently, Borg *et al.* reported a crucial role of CD73 on T-cells in the cardiac wound healing process and ventricular remodeling post-I/R 23. However, the contribution and mechanism underlying the action of CD73^+^Tregs in cardiac repair post-MI remains elusive.

In the present study, we investigated the contribution of CD73 on Tregs to the resolution of cardiac inflammation and remodeling after permanent infarction and evaluated the efficacy of the IL2C in expanding CD73^+^Tregs as well as in attenuating cardiac dysfunction.

## Materials and Methods

### Animal studies

The present study used 8-10-week-old male mice with a C57BL/6 background. All animal procedures were performed in accordance with the Institutional Animal Care and Use of Laboratory Animals approved by the Tongji University Institutional Animal Care and Use Committee (TJLAC-017-025). WT mice were obtained from the Shanghai Laboratory Animal Co., Ltd. (SLAC, Shanghai, China). CD73 knockout (KO) mice were purchased from the Jackson Laboratory (Bar Harbor, ME). Forkhead box P3 (FoxP3) -YFP knock-in mice were kindly provided by Professor Bin Li (Shanghai Institute of Immunology & Department of Immunology and Microbiology, Shanghai JiaoTong University School of Medicine, China) 24.

Mice were anaesthetized with pentobarbital sodium (50 mg/kg, intraperitoneal injection) for one time, and mechanically ventilated (isoflurane 1-2% vol/vol) with an Inspira - Advanced Safety Ventilator (Harvard Apparatus). Then murine MI models were set up by left anterior descending coronary artery (LAD) ligation, as previously reported 6. The mice were euthanized with CO_2_ gas for further experiments.

Detailed methods are provided in the supplementary material online.

### Human studies

Blood samples were collected from patients who underwent acute myocardial infarction (AMI) (n = 36) up to 7 days after the hospitalization. Age-matched non-MI patients (n = 24) with chest pain according to their clinical diagnosis from the Department of Cardiology of Shanghai East Hospital were used as control. This study was approved by the Institutional Ethics Committee of Shanghai East Hospital, Tongji University School of Medicine (No. ECSEH2019-004). All enrolled patients provided written informed consent. More detailed information is provided in the supplementary material online, and the baseline characteristics of all subjects are listed in **Table S1**.

### Statistical analysis

All data are presented as mean ± standard error of the mean (SEM). All data were checked for normality and equal variance before analysis by Shapiro-Wilk test, and then analyzed by SPSS 11.0 statistical software (SPSS Inc., USA) and GraphPad Prism 8 statistical software (GraphPad Software Inc, San Diego, CA). Comparisons between two groups were analyzed by unpaired Student's t-test. One-way ANOVA with Tukey post hoc tests was used for comparisons between multiple groups; and two-way ANOVA was used for comparisons between multiple groups when there were 2 experimental factors. For comparison of composition ratios in clinical data, Pearson's chi-squared test or, if not suitable, Yates' corrected chi-squared test was performed. Spearman's rank correlation was used to assess the relationship between the level of NT-pro BNP, troponin, myoglobin, creatine kinase isoenzyme (CKI), and the proportion of CD4^+^CD73^+^ cells in Peripheral blood mononuclear cells (PBMCs) in patients. Logistic regression model was set up to show the relationship between the percentage of CD73 in CD4^+^T cells and MI, and the percentage of CD73 in Tregs and MI. Models also adjusted by age, gender, body mass index (BMI), systolic blood pressure value, diastolic blood pressure value, total cholesterol, triglyceride, low density lipoprotein, high density lipoprotein and fasting blood glucose. *P* value of <0.05 was considered as statistical significance.

## Results

### MI elicits the activation of the purinergic signaling cascade in cardiac mononuclear cells and Tregs

**Figure 1:**
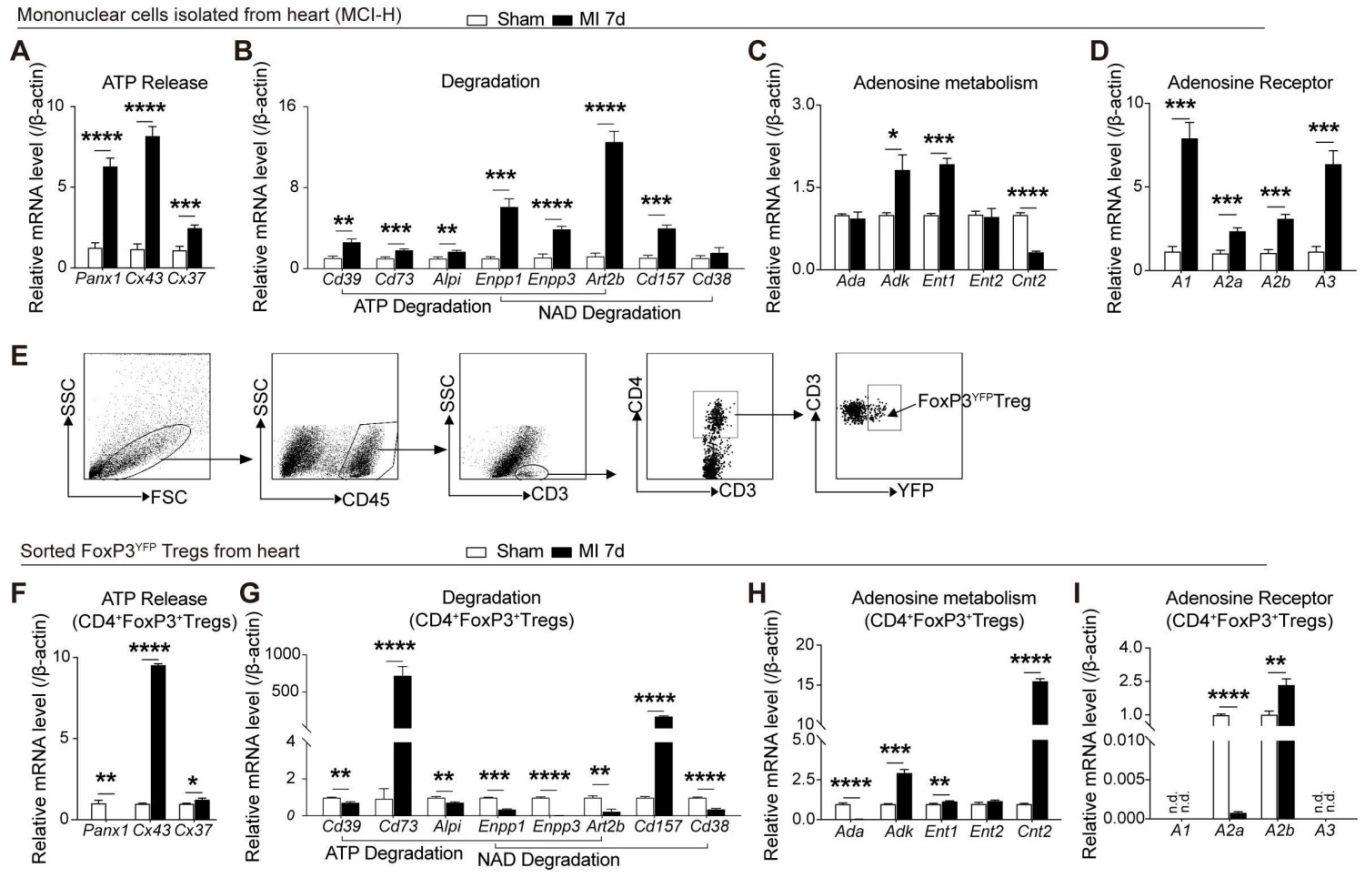
** MI elicits the activation of the purinergic signaling cascade in cardiac mononuclear cells and Tregs. A-D,** Mononuclear cells isolated from hearts (MCI-H) after digestion at 7-day post MI in sham and MI group, and the mRNA expression levels of factors related to ATP release **(A)**, ATP and NAD degradation **(B)**, adenosine metabolism **(C)** and adenosine receptor **(D)** in MCI-H, normalized to β-actin. **E,** Sorting strategy for CD45^+^CD3^+^CD4^+^FoxP3^YFP^ cells (Tregs) in MCI-H from the murine heart. **F-I,** mRNA levels of factors related to ATP release **(F)**, ATP and NAD degradation **(G)**, adenosine metabolism **(H)** and adenosine receptor **(I)** in sorted FoxP3^+^Tregs from murine heart, normalized to β-actin. Sorted FoxP3^+^ Tregs and FoxP3^-^ cells were sorted from the heart from 3-4 pooled MI mice and 5-6 pooled sham mice. Data are mean ± SEM. MCI-H indicates mononuclear cells isolated from hearts; ATP, adenosine triphosphate; NAD, nicotinamide adenine dinucleotide; n.d., not detected; Tregs, regulatory T cells. *P < 0.05, **P < 0.01, ***P < 0.001, **** P < 0.0001.

Previous studies showed the distinction of purinergic signaling in cardiac T cells from peripheral T cells 23, and increased Tregs peaking on day 7 after MI^5^, ^25^. To determine whether cardiac infarction triggered the activation of purinergic signaling on cardiac mononuclear cells, murine infarcted and sham hearts were digested after 7 days following MI for further examination. As depicted in **Figure 1A**, mRNA levels of *Pannexin* (*Panx)1*, *connexin(Cx)43*, and *Cx37,* which are related to release of ATP and NAD 26, were upregulated in the mononuclear cells isolated from heart (MCI-H) from the MI group. Enzymes related to the decomposition of ATP and NAD also increased in the MCI-H from the MI group (**Figure 1B**). Similarly, expressions of *Adk* and *Ent1* were elevated in the MI mice compared to that in the sham (**Figure 1C**). Substantial amounts of ATP are eventually hydrolyzed into adenosine, which binds to different adenosine receptors to exert its activities. Expression of adenosine receptors including *A1*, *A2a*, *A2b*, and *A3* were all increased in MCI-H from the MI group compared to that from the sham group (**Figure 1D**).

A recent report confirmed a phenotypically and functionally unique population of heart Tregs 27. Therefore, we evaluated the purinergic signaling in cardiac CD4^+^FoxP3^YFP^ Tregs sorted from the injured and sham hearts (**Figure 1E**). Interestingly, the mRNA levels of *Panx1* decreased in Tregs from MI heart (**Figure 1F**), which differed from the mRNA profile associated with purinergic pathway in MCI-H (**Figure 1A**). In addition, the expressions of *Cd73* and *Cd157* increased dramatically (about 800- and 200-fold, respectively) in the MI group compared to the sham (**Figure 1G**). Contrast to the lower *Cnt2* expression in the MCI-H of MI group (**Figure 1C**), *Cnt2* mRNA levels were upregulated strikingly in the cardiac Tregs in MI group compared to those in the sham group (**Figure 1H**). Furthermore, *A2a* mRNA expression decreased, whereas *A2b* increased in Tregs from MI hearts (**Figure 1I**). These results indicated that cardiac Tregs underwent purinergic metabolic reprogramming after permanent ligation of the LAD, which may be involved in its functional changes.

### Tregs infiltrate into the injured heart and highly express CD73 post MI

**Figure 2:**
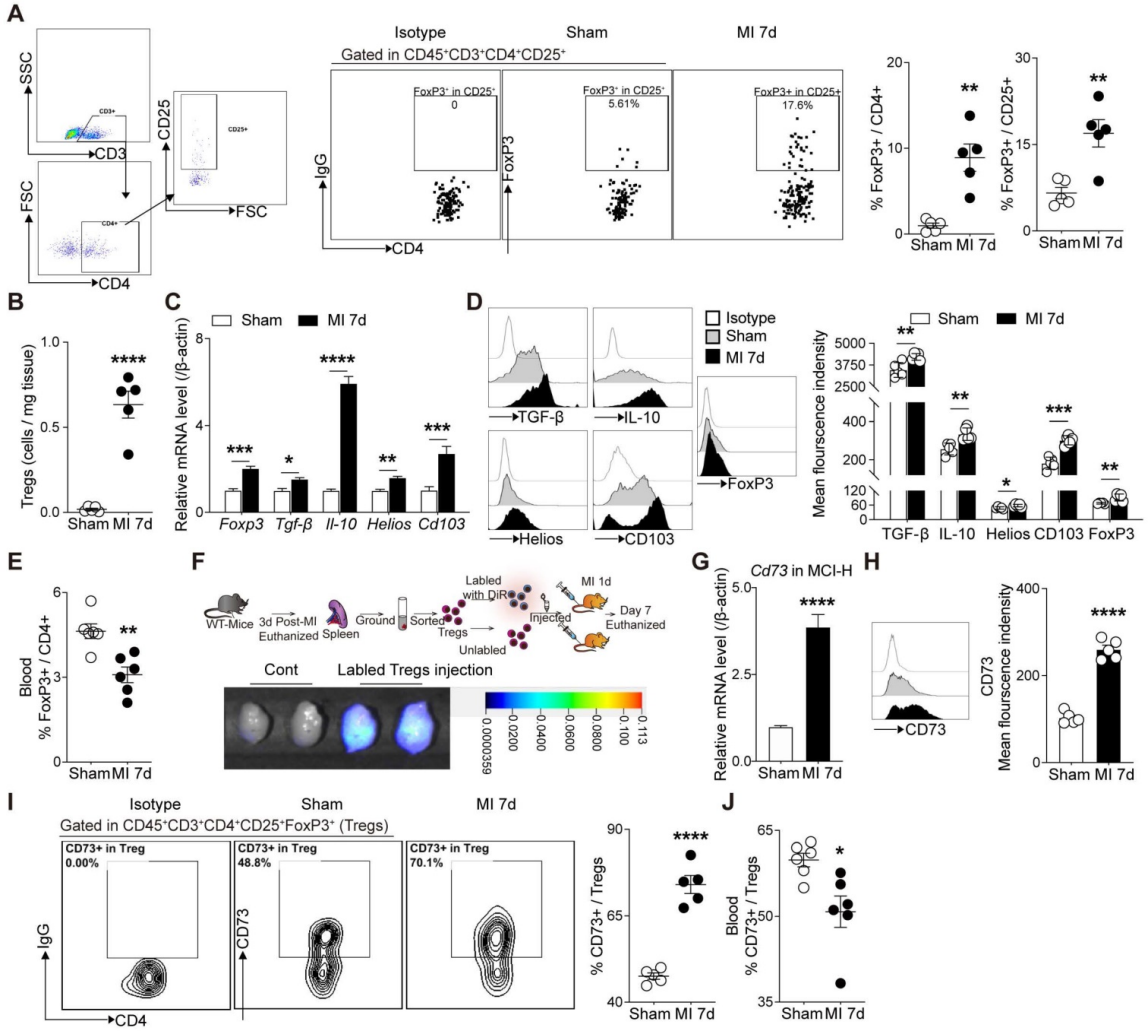
** Tregs infiltrate into the injured heart and highly express CD73 post MI. A,** Analysis strategy, representative flow cytometry dot plot and percentages of FoxP3^+^ in CD4^+^ cells in the heart (n = 5) and the percentages of FoxP3^+^ gated in CD4^+^CD25^+^ cells (n = 5). **B,** The number of Tregs in the heart (n = 5). **C-D,** FoxP3, TGF-β, IL-10, Helios and CD103 mRNA levels (**C**), representative flow cytometry histogram and mean fluorescence intensity in MCI-H post MI compared to the control (**D**). **E,** Percentage of FoxP3^+^ in CD4^+^ cells in the blood (n = 5). **F,** Schematic diagram of an in vivo experiment for cell tracing and the heart Imaging results of DiR-labeled CD4^+^CD25^+^ Tregs infusion. **G-H,** CD73 mRNA level (**G**), representative flow cytometry histogram and mean fluorescence intensity in MCI-H post MI compared to the control(**H**).** I,** Representative flow cytometry contour plot and the percentages of CD73 in CD45^+^CD3^+^CD4^+^CD25^+^FoxP3^+^ cells (Tregs) in the heart (n = 5). **J,** The percentage of CD73 in Tregs in the blood (n = 5). Data are mean ± SEM. MCI-H indicates mononuclear cells isolated from hearts; Tregs, regulatory T cells. *P < 0.05, **P < 0.01, ***P < 0.001, **** P < 0.0001.

Next, we sought to confirm whether cardiac Tregs were accumulated from peripheral tissues post MI. The frequency of Tregs in heart, mediastinal lymph nodes (MLN), blood, and spleen were evaluated by flow cytometry. As shown in **Figure 2A**-**B**, the percentage of FoxP3^+^ Tregs gated from CD4^+^ cells and the absolute number of CD4^+^FoxP3^+^ cells were both increased in MI group compared to the sham group. Also, marked elevations of Tregs-related markers, including FoxP3, TGF-β, IL-10, Helios, and CD103, in MCI-H were shown in MI group compared to the sham (**Figure 2C-D**). Flow results showed that the percentage of Tregs increased in the heart-draining lymph nodes—MLN, while decreased in the blood and spleen in MI group compared to the sham (**Figure 2E, S1A-B**). To further monitor the source of cardiac Tregs cells, sorted Tregs were labeled with DiR, then transferred to the recipient mice. After 7 days, numerous DiR-labeled CD4^+^CD25^+^ cells were found in the infarcted heart (**Figure 2F**). In concordance, Tregs cells from FoxP3-YFP knock in mice (sham) were isolated and transferred to recipient MI mice (without YFP on FoxP3), and approximately half of FoxP3^+^ Tregs expressed YFP in the injured hearts (**Figure S1C**). Furthermore, the expressions of chemokines and chemokine receptors were all upregulated in the peri-infarct area of the MI heart (**Figure S1D-E**), which supported the chemotaxis activity of T-cells including Tregs 28, 29.

Considering the striking upregulation of *Cd73* mRNA level in cardiac Tregs after MI, we then validated the CD73 levels in different immune cell populations and the cardiac regions. CD73 levels increased dramatically in the peri-infarcted heart tissue (**Figure S2A**) and MCI-H (**Figure 2G-H**). Importantly, *Cd73* expression in infarcted cardiac Tregs was much higher than that in effector T-cells (Teffs) (**Figure S2B**), although the percentage of CD73 gated on CD4^+^ cell and CD8^+^ cell also increased after MI (**Figure S2C-D**). Furthermore, infarcted cardiac FoxP3^+^ Tregs expressed considerably more CD73 than the sham group (**Figure 2I**). Interestingly, CD73 in Tregs from blood was decreased in MI group compared to the sham (**Figure 2J, S2E**). Collectively, MI induced a great degree of Tregs infiltration into the injured hearts where they express high levels of CD73.

### CD73 deficiency impairs the protective function of Tregs in cardiac healing post-MI

**Figure 3:**
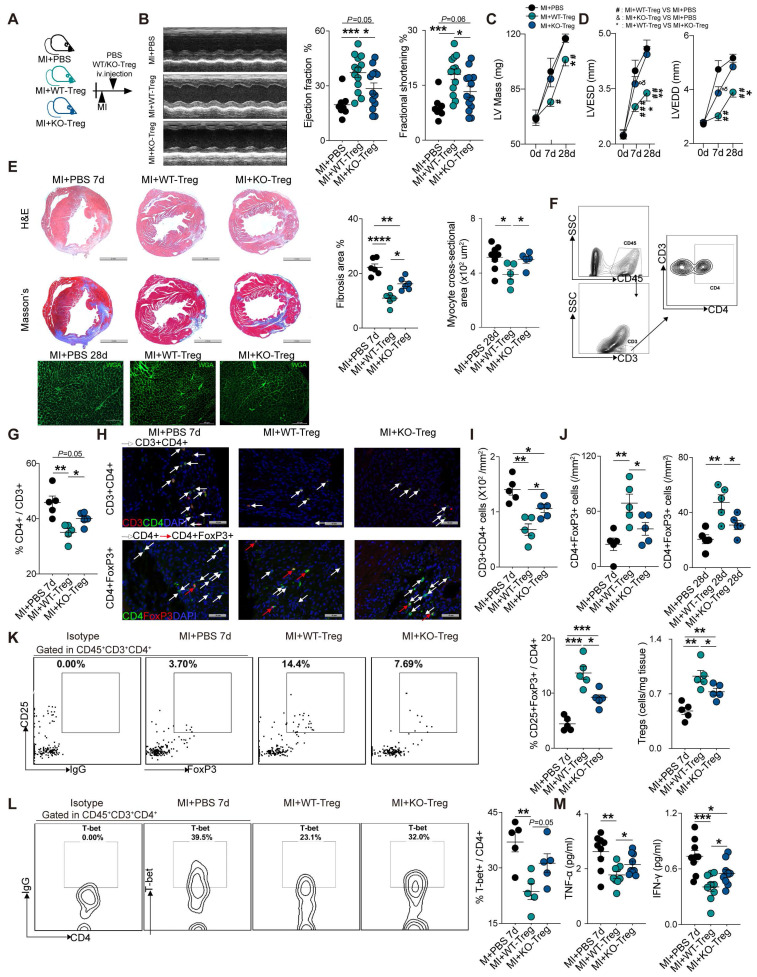
** CD73 deficiency impairs the protective function of Tregs in cardiac healing post-MI. A,** Schematic diagram of an in vivo experiment for cell transfer experiments to compare the effect of CD73KO-Tregs with WT-Tregs post-MI. **B-D**, representative images, ejection fraction and fractional shortening **(B)**, LV Mass **(C)** and LVESD/LVEDD **(D)** by echocardiography in MI+PBS, MI+WT-Tregs and MI+CD73KO-Tregs transferred mice (n>5 in each group). **E,** Representative image of H&E, Masson staining, the percentage of fibrosis area (n = 6, Scale bars: 2 mm), and myocyte cross-sectional area by wheat germ agglutinin (WGA) staining (n≥5, Scale bars: 100 µm). **F-G,** Analysis strategy and the percentage of CD4^+^ in CD45^+^CD3^+^ cells in MCI-H after administration (n = 5). **H-J,** Representative Immunofluorescence staining, and the number of CD3^+^CD4^+^ T cells and CD4^+^FoxP3^+^ Tregs in the peri-infarct areas of heart (n = 5) in each group. Scale bars: 50 µm. White arrows represent CD4^+^ cells and red arrows represent CD4^+^FoxP3^+^ cells. **K,** Representative flow cytometry dot plot and percentages of CD25^+^FoxP3^+^ in CD45^+^CD3^+^CD4^+^ cells in MCI-H after administration (n = 5) in each group, and the number of Tregs in the heart (n = 5). **L,** Representative flow cytometry contour and percentages of T-bet^+^ in CD45^+^CD3^+^CD4^+^ cells in MCI-H isolated from each group mice (n = 5). **M,** The protein levels of TNF-α and IFN-γ in the peri-infarct areas of heart tissue (n = 9). Data are mean ± SEM. LVESD indicates left ventricular end-systolic diameter: LVEDD, left ventricular end-diastolic diameter; Tregs, regulatory T cells. *P < 0.05, **P < 0.01, ***P < 0.001; **** P < 0.0001; **C-D**, ^#,^ MI+WT-Treg group compared with MI+PBS group; *, MI+WT-Treg group compared with MI+KO-Treg group; ^&^, MI+KO-Treg group compared with MI+PBS group.

To further investigate the role of CD73 on Tregs in heart healing, CD4^+^Tregs were sorted from WT and CD73KO mice (**Figure S3A-C**), followed by adoptive transfer into WT recipient mice at day 1 post-MI (**Figure 3A**). At day 7, administration of WT-Tregs improved the ejection fraction (EF) and fractional shortening (FS) while CD73KO Tregs treatment did not when compared with MI-PBS groups (**Figure 3B**). Furthermore, CD73KO Tregs showed no efficacy on the LV remodeling after 28 days post-MI [increased LV mass (**Figure 3C**), LV end-diastolic diameter (LVEDD) and LV end-systolic diameter (LVESD) (**Figure 3D**)], while WT Tregs protected the heart from remodeling. More severe cardiac fibrosis and larger cardiomyocytes were also found in CD73KO Tregs treatment group post-MI (**Figure 3E**). Moreover, animals treated with CD73KO Tregs had a higher percentage of CD4^+^ gated in CD3^+^ cells, higher count of CD3^+^CD4^+^ T cells, a lower percentage of CD25^+^FoxP3^+^ gated in CD4^+^ cells, and lower count of CD4^+^FoxP3^+^ Tregs in the heart compared to the WT Tregs treatment (**Figure 3F-K**). Also, the increased Tregs in infarcted heart maintained the higher level until at least 28 days in WT-Tregs group compared with the KO-Tregs group (**Fig. S3D**), which suggested that CD73 on Tregs are necessary for their selective recruitment to the injured heart. Furthermore, WT-Tregs could reduce T-bet^+^ T cell infiltration in the heart while CD73KO Tregs showed less efficiency (**Figure 3L**). Also, higher protein levels of TNF-α and IFN-γ, which are mainly secreted by Th1, were found in the peri-infarct area in mice treated with CD73-deficient Tregs (**Figure 3M**). Collectively, CD73 deficiency attenuated the protective function of Tregs in cardiac healing post-MI.

### Deficiency of CD73 on Tregs displays decreased tissue tropism and immunosuppressive function

**Figure 4:**
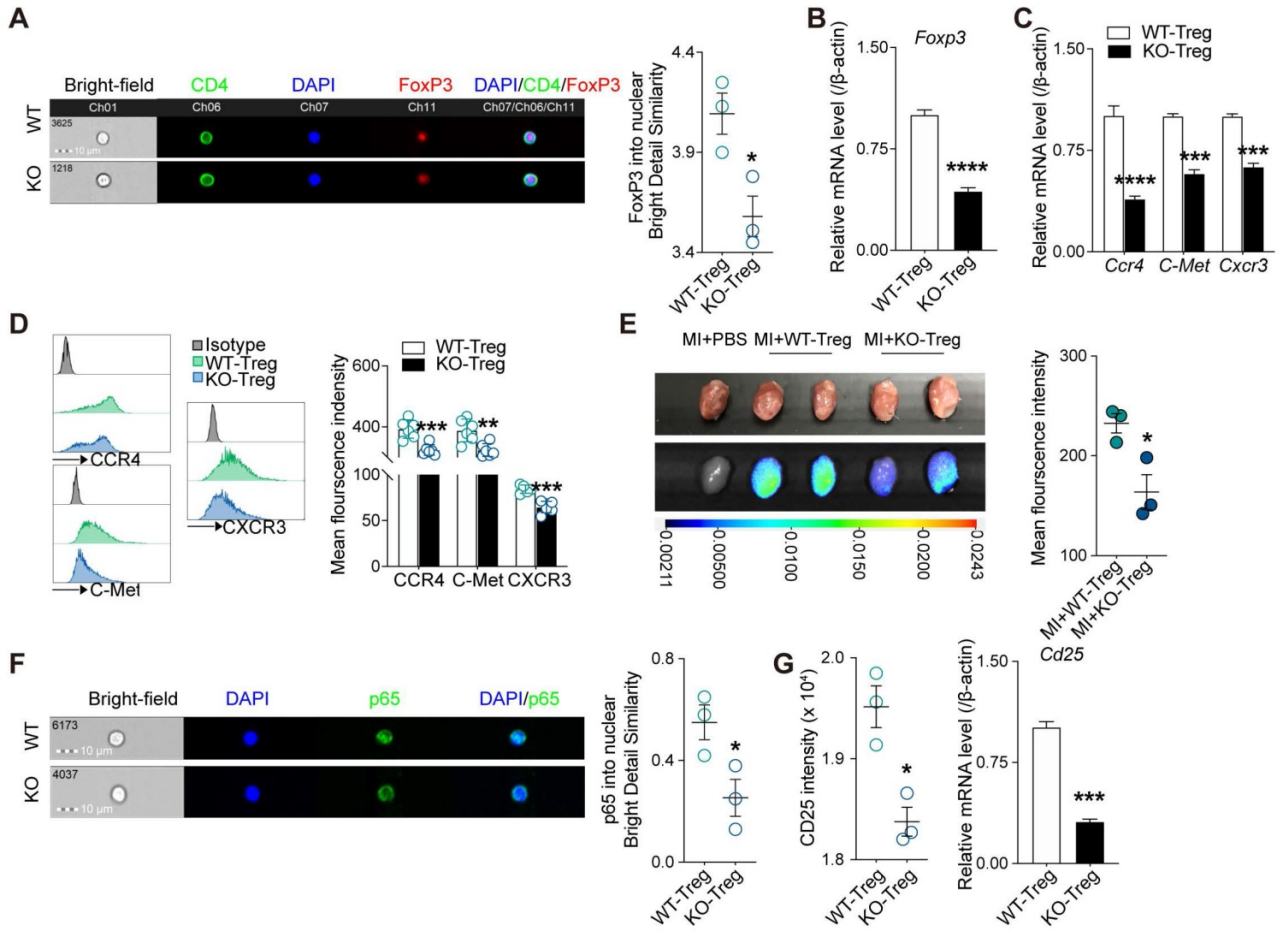
** Deficiency of CD73 in Tregs displays decreased tissue tropism and immunosuppressive function.** CD4^+^CD25^+^ Tregs were sorted from spleen in WT and CD73 KO mice.** A,** Representative ImageStream pictures (left) and mean similarity scores (right) for the colocalization of FoxP3 and DAPI in WT/KO CD4^+^CD25^+^ Tregs under basal conditions. **B,** FoxP3 mRNA level in WT/KO CD4^+^CD25^+^ Tregs. **C-D,** CCR4, C-Met, CXCR3 mRNA level (**C**) and representative flow cytometry histogram and mean fluorescence intensity (**D**) in WT/KO CD4^+^CD25^+^ Tregs. **E,** Imaging results of labeled CD4^+^CD25^+^ Tregs infusion and tracing after perfusing. **F,** Representative ImageStream pictures (left) and mean similarity scores (right) for the colocalization of P65 and DAPI in WT/KO CD4^+^CD25^+^ Tregs under basal conditions.** G,** the intensity of CD25 and the mRNA level of *Cd25* in WT/CD73KO CD4^+^CD25^+^ Tregs. Tregs indicates regulatory T cells. Data are mean ± SEM. *P < 0.05, **P < 0.01, ***P < 0.001, ****P < 0.0001. **A, E, and F,** n = 3 independent experiments.

Given adenosine promotes FoxP3 expression 30 and lack of CD73 affects adenosine production 31, we next sought to determine whether deficiency of CD73 affects the phenotype and function of Tregs. As the results from imaging flow cytometry showed, Tregs deficient in CD73 decreased nuclear levels of FoxP3 under basal conditions (**Figure 4A**), and the mRNAs levels of *Foxp3* in CD73KO Tregs (**Figure 4B**). Tissue tropism is important for Tregs to exert their function locally. Hence, we firstly measured genes involved in Tregs recruitment (CCR4, C-Met, and CXCR3) and found decreased gene and protein levels in CD73KO Tregs (**Figure 4C-D**). Next, we labeled Tregs with DiR and transferred to recipient mice following MI, then found lower mean fluorescence intensity in the injured heart in the CD73KO-Tregs treatment group than in the WT-Tregs group (**Figure 4E**). In addition, a lower CD4^+^FoxP3^+^ Tregs count and percentage of CD25^+^FoxP3^+^ gated in CD4^+^ cells were found in the heart in the CD73KO-Tregs treatment group compared to the WT-Tregs group (**Figure 3H-J, S3D**). These results indicated weakened tissue tropism in CD73KO Tregs.

The nuclear levels of NF-κB subunit p65, which is essential for Treg-dependent maintenance of immune tolerance 32, was decreased in CD73KO Tregs (**Figure 4F**). Moreover, CD73-deficient Tregs expressed lower intensity level and mRNA level of *Cd25* (**Figure 4G**). Also, we found that CD73KO-Tregs have weakened suppressive function in inhibiting the proliferation of Teffs in coculture system compared with WT-Tregs (**Figure S4A**). Therefore, the decreased suppressive activity of CD73KO-Tregs may be attributed to their reduced nuclear levels of FoxP3 and p65, which trigger downregulated CD25 expression and other molecules mediating their suppression of Teffs.

### CD73 derived from CD4^+^FoxP3^+^Tregs can bind to CD4^+^FoxP3^-^ Teffs and inhibit their inflammatory response

**Figure 5:**
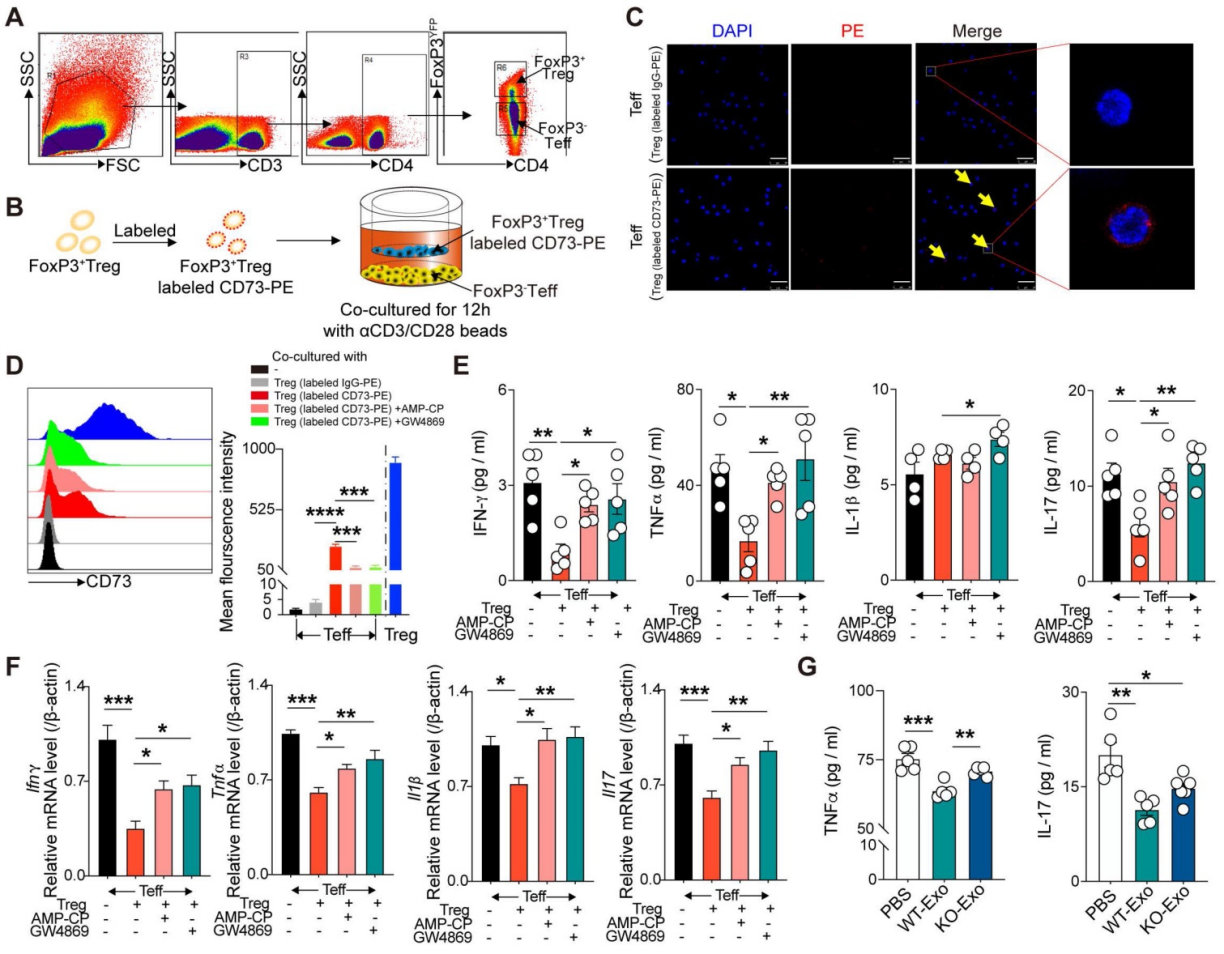
** CD73 derived from CD3^+^CD4^+^FoxP3^+^Treg cells can bind CD4^+^FoxP3^-^ Teff cells and inhibit the inflammatory response of T effectors. A,** sorting strategy for CD3^+^CD4^+^CD25^+^FoxP3^YFP^ cells sorted from splenetic single cell suspension in FoxP3-YFP mice.** B,** Schematic diagram of an in vitro co-cultured experiment to investigate the interaction between FoxP3^+^Treg and FoxP3^-^Teff. **C,** CD73 banded on the surface of Teffs cells by confocal microscopy. Scale bars: 25μm.** D,** Representative flow cytometry histogram and mean fluorescence intensity of CD73 in different treated Teffs or Tregs. **E-F,** Quantification of IL-1β, TNF-α, IFN-γ, and IL-17 secreted protein level (**E**) and mRNA level (**F**) of Teffs after co-cultured. **G,** Quantification of TNF-α and IL-17 secreted protein level of Teffs after treatment with WT/KO-Tregs derived-exosomes. Data are mean ± SEM. GW4689, an inhibitor of exosome biogenesis/release; Tregs, regulatory T cells; Teff, effector T-cell. *P < 0.05, **P < 0.01, ***P < 0.001, ****P < 0.0001.

To determine the mechanism by which CD73 on Tregs affected Teffs, FoxP3^+^ Tregs and FoxP3^-^ Teffs were sorted from the spleen of FoxP3-YFP mice and then cocultured in 1-µm transwells after labeling Tregs with CD73-PE (**Figure 5A-B**). After 12h, confocal microscopy results revealed the binding of Treg-derived CD73 on the surface of Teffs (**Figure 5C**). Flow cytometry further confirmed higher level of CD73 mean fluorescent intensity (MFI) on FoxP3-Teffs, and addition of adenosine 5'-(α, b-methylene) diphosphate (AMP-CP, a CD73 inhibitor) significantly reversed this elevation (**Figure 5D**). A previous study found that CD73-expressing exosomes produced by Tregs contributed to their suppressive activity in a murine Treg-cell line with self-specificity (Auto-Treg cells) 33. In the present coculture system, addition of GW4869 (an exosome inhibitor) further decreased the expression of CD73 derived from Tregs on FoxP3-Teffs (**Figure 5D**). Moreover, coculture with FoxP3^+^Tregs also downregulated the protein and mRNA levels of IL-1β, TNF-α, IFNγ, and IL-17 in Teffs, whereas addition of AMP-CP or GW4869 partly restored the expressions of TNF-α, IFNγ, and IL-17(**Figure 5E-F**). Also, we found WT-Tregs-derived exosomes could sufficiently decrease the production of TNF-α and IL-17 by Teffs, while CD73KO-Tregs-derived exosomes couldn't (**Figure 5G**). Collectively, these results suggested that Tregs-derived CD73 can bind to the surface of Teffs and exert a regulatory effect, and this process may be partly mediated by Tregs-derived exosomes.

### Administration of low-dose of IL-2/anti-IL-2 complex resulted in FoxP3^+^CD73^+^ Tregs expansion in the heart and contributed to the recovery of cardiac function

**Figure 6:**
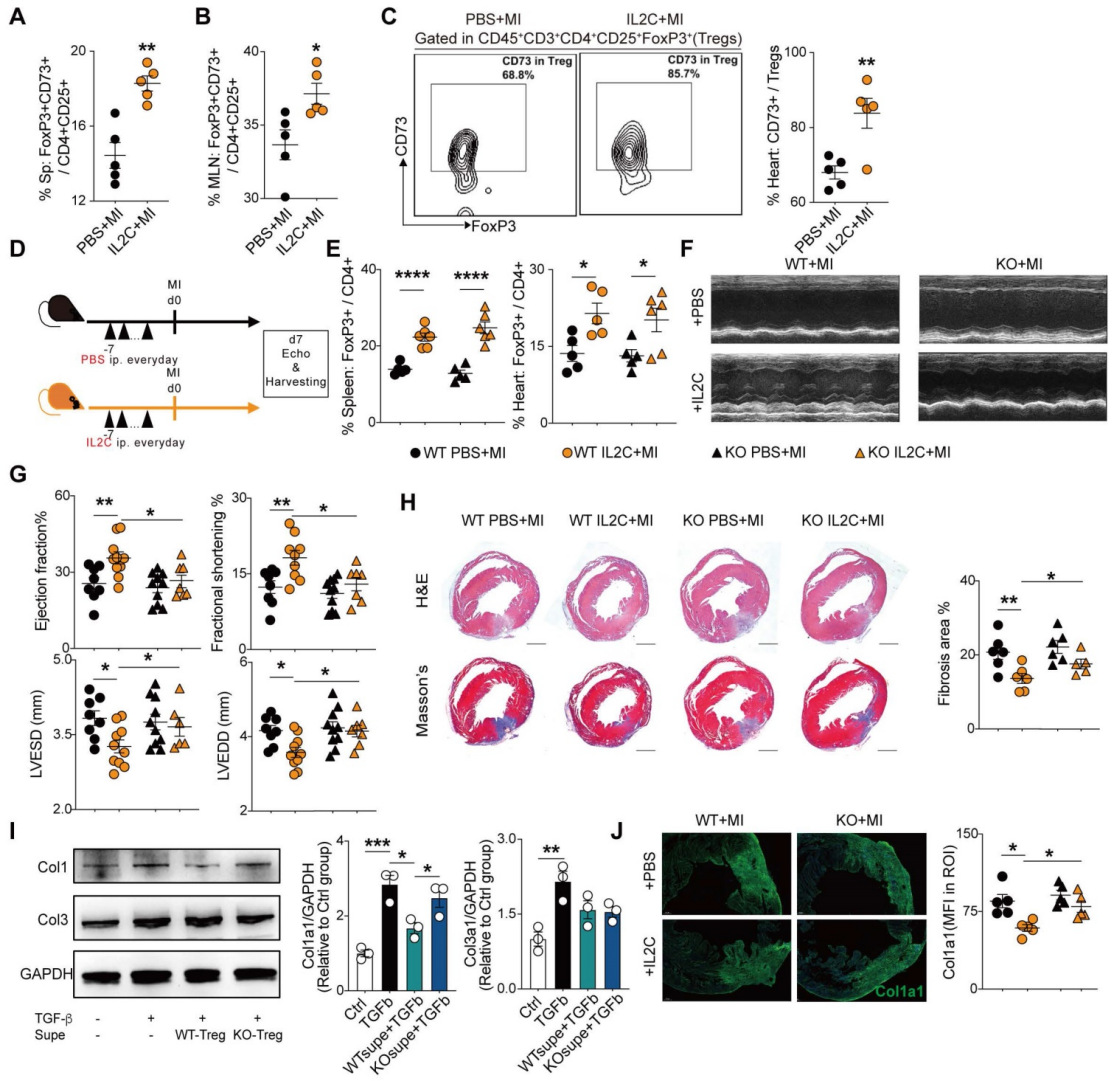
**Administration of low-dose of IL-2/anti-IL-2 complex resulted in FoxP3^+^CD73^+^ Tregs expansion in the heart and contributed to the recovery of cardiac function. A-B,** Percentages of FoxP3^+^CD73^+^ cells in CD4^+^CD25^+^ cells in Spleen **(A)** and in MLN **(B)** (n = 5). **C,** Representative flow cytometry contour plot and percentages of CD73^+^ gated in CD45^+^CD3^+^CD4^+^CD25^+^FoxP3^+^ cells (Tregs) in the heart (n = 5). **D,** Schematic diagram of in vivo experiment. **E,** The percentage of FoxP3^+^ cell gated on CD4^+^ cell in spleen and heart after IL2C treatment(n>5 in each group). **F-G,** representative images, ejection fraction, fractional shortening, LVESD, and LVEDD at day 7 post-MI by echocardiography in IL2C treated WT/KO mice (n>6 in each group). **H,** Representative image of H&E, Masson staining, the percentage of fibrosis area (n>5, scale bars: 1mm). **I,** the protein level of Col1a1, Col3a1 in cardiac fibroblasts with/without WT/KO-Treg Supe treatment. **J,** Representative Immunofluorescence staining, and mean fluorescence intensity of Col1a1 in the injured areas of heart (n = 5) in each group. Scale bars: 200 µm. IL2C indicates IL-2/anti-IL-2 complex; Sp, Spleen; MLN, mediastinal lymph nodes; mean fluorescence intensity, MFI; ROI: Region of interest; Tregs, regulatory T cells. *P < 0.05, **P < 0.01, ***P < 0.001, ****P < 0.0001.

Previous studies demonstrated that the expansion of Tregs with low dose IL2C attenuated HF progression 34 and cardiac ischemia-reperfusion injury 35. In accordance, we also found that IL2C could improve cardiac function and ventricular remodeling post-MI. Moreover, we sought to explore whether IL2C can expand CD73^+^Tregs in murine hearts post-MI. Mice were randomly allocated into the IL2C- or PBS-treated groups (**Figure S5A**). Flow cytometry results revealed that administration of IL2C significantly upregulated the percentages of CD4^+^CD73^+^ T cells among CD3^+^ T cells of the spleen, MLN, and heart compared to that in the MI control (**Figure S5B**). Furthermore, the ratio of CD73^+^FoxP3^+^ Tregs among CD4^+^CD25^+^ cells also increased in the spleen and MLN post treatment (**Figure 6A-B, S5C**). Moreover, cardiac Tregs presented a higher expression of CD73 when treated with the IL2C (**Figure 6C**). To further confirm the role of CD73 in cardiac recovery by IL2C induced Tregs expansion, WT and CD73KO mice were treated with IL2C before surgery (**Figure 6D**). We found that administration of IL2C markedly increased the frequency of CD4^+^FoxP3^+^Tregs in the spleen and heart in both WT and KO groups (**Figure 6E**). However, heart function (evaluated by EF, FS, LVESD, and LVEDD by echocardiography) were greatly improved in WT mice subjected to MI after IL2C treatment but not in KOs when compared with PBS treated group post-MI (**Figure 6F-G, S5D**). We also found less cardiac fibrosis in WT group after IL2C treatment when compared with IL2C treated KO mice (**Figure 6H**). In addition, low doses of IL2C decreased the mRNA levels of *Ifn-γ, Il-1β, Tnf-α, Mmp2, α-Sma, Anp, Bnp, and Bcl2/Bax* in infarcted hearts in the WT group (**Figure S5E**).

Then, to further explore whether CD73^+^Tregs could affect fibroblasts, WT or CD73KO-Tregs supernatant was used to treat fibroblast, and we found that supernatant from WT-Tregs but not CD73KO-Tregs could ameliorate TGFβ-induced Col1a1 expression, while there is not difference in Col3a1 level (**Figure 6I**). Further results revealed that IL2C treatment significantly decreased the level of Col1a1 in the injured heart in WT mice but not CD73KO mice after MI (**Figure 6J**). Therefore, the IL2C caused FoxP3^+^CD73^+^Tregs expansion in murine MI models, which contributes to cardiac healing post-MI.

### CD73 expression on CD4^+^ T cells in PBMCs were negatively correlated with the NT pro-BNP and myocardial zymogram levels in serum in patients with AMI

**Figure 7:**
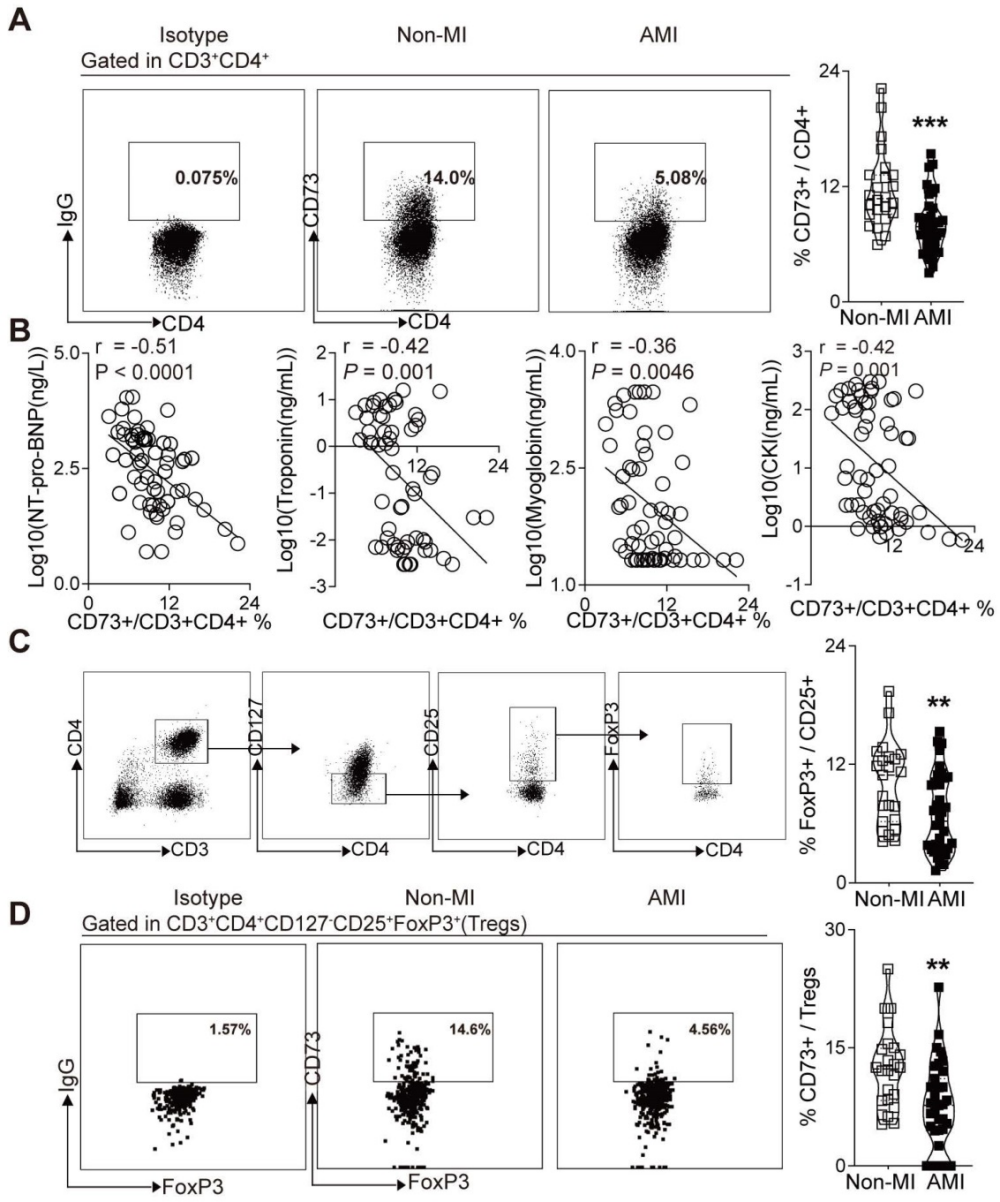
** Decreased expression of CD73 on CD4+ T cells and Tregs in PBMCs is associated with increased percentage of AMI. A,** Representative flow cytometry dot plots and percentage of CD73^+^ gated in CD3^+^CD4^+^ T cells in PBMC from AMI and non-MI patients. **B,** Correlation analysis of the NT pro-BNP, Troponin, Myoglobin, CKI levels and the ratio of CD73^+^ in CD3^+^CD4^+^ cells in PBMC from AMI and non-MI patients. Line represents linear regression of data. Sites with coefficients, and *P* values inside plots. **C-D,** Analysis strategy, representative flow cytometry dot plots and percentage of positive cells in PBMC from AMI and non-MI patients, for FoxP3^+^ in CD3^+^CD4^+^CD127^-^CD25^+^ cells subsets **(C)** and for CD73^+^ on CD3^+^CD4^+^CD127^-^CD25^+^FoxP3^+^ cells (Tregs) **(D).** Data are mean ± SEM. AMI indicates acute myocardial infarction; PBMC, peripheral blood mononuclear cell; NT pro-BNP, N-terminal pro-brain natriuretic peptide; CKI, creatine kinase isoenzyme; Tregs, regulatory T cells; OR odds ratio. **P < 0.01; ***P < 0.001.

Based on the crucial role of CD73 on T cells in the cardiac wound healing after I/R injury^23^ and the vital function of CD73^+^ Tregs in our murine MI models, we sought to determine whether human CD73 on CD4^+^ T cells and Tregs is involved in cardiac healing in patients subjected to AMI. Peripheral blood samples were collected from subjects with AMI and control subjects, matched by age and sex. Demographic and clinical characteristics are listed in **Table S1**. Similar to our murine study (**Figure 2J**), flow cytometry analysis revealed a lower percentage of CD73 on CD4^+^ T cells in PBMCs of AMI patients compared to patients without MI (**Figure 7A**). Moreover, correlation analysis revealed that CD73 expressions on CD4^+^ T cells were negatively correlated with the serum levels of NT pro-BNP, troponin, myoglobin, CKI (**Figure 7B**), and EF value (**Figure S6A**). Next, we applied logistic regression model to assess the relationship between the percentage of CD73 on CD4^+^T cells and MI prevalence. Our results showed that in lower level of CD73^+^/CD4^+^ cells group, MI ratio is 4-fold higher (7.663-fold after being adjusted) than that in higher level group (**Figure S6B, Table S4**). Moreover, the frequency of Tregs (identified as CD3^+^CD4^+^CD127^-^CD25^+^FoxP3^+^ T cells in humans) was reduced in PBMCs of AMI patients (**Figure 7C**). CD73 expression on Tregs was also downregulated in PBMCs from AMI patients compared to those in control patients (**Figure 7D**). And the logistic regression model showed that the ratio of MI in people with lower level of CD73^+^/FoxP3^+^Tregs is 4.333-fold higher (11.043-fold after being adjusted) than that in high level group (**Figure S6C, Table S5**). These results indicated that, in patients subjected to AMI, CD73 on CD4^+^ T cells and Tregs may be involved in cardiac healing.

## Discussion

The expression of the ectoenzyme CD73 on T cells was reported to be crucial in the cardiac healing process in TAC-Induced HF and in cardiac I/R injury 22, 23. In the present study, we found dramatic increase of CD73 expression in infarcted cardiac Tregs than Teffs post MI. Further results confirmed that MI contributed to the activation of purinergic signaling in cardiac Tregs and CD73 deficiency decreased the protective function of Tregs in cardiac healing post-MI. Importantly, we demonstrated the negative correlation between the percentage of peripheral blood CD73 gated in CD4^+^ T cells and the levels of indicators of myocardial injury in serum of AMI patients. These results highlighted the importance of CD73^+^FoxP3^+^Tregs in inflammation resolution and cardiac healing post-MI.

Following MI, a variety of inflammatory cells accumulate into the infarcted region. Excessive inflammation, poor healing response, and ventricular remodeling contribute to the cardiac dysfunction post-MI. Recent studies confirmed the importance of T cells in cardiac inflammation and ventricular remodeling after injury 2, 5, 23, 36. The pivotal role of purinergic signaling in T cell functions and responses was also evaluated in TAC-induced HF model and cardiac I/R injury model recently 22, 23. Compared to the circulating CD4^+^T cells, cardiac CD4^+^T cells underwent purinergic metabolic reprogramming, which are responsible for the accelerated release and hydrolysis of ATP, cAMP, AMP, and NAD to adenosine 37. Our data from the sham and MI hearts indicated that infarction markedly triggered the activation of the purinergic signaling in MCI-H. Since Tregs are known as a key mediator in blocking the excessive inflammation and tissue injury and a recent report confirmed a phenotypically and functionally unique population of heart Tregs 27, we evaluated the purinergic metabolic reprogramming in cardiac Tregs post-MI. Our results revealed that *Cd73* (about 800-folds) and *Cd157* (about 200-folds) expression increased greatly in mRNA level in purified FoxP3^+^Tregs from MI heart compared to that from the sham, and higher expression of *Cd73* was found in Tregs than Teffs isolated from those infarcted heart. This striking upregulation further highlighted the importance of CD73 on Tregs in heart post-MI. Previously, NAD degrading enzyme CD157, expressed on CD56(bright)CD16(-) NK cells, had been reported to have regulatory activities 38. Our further results revealed that *Cd157* is higher in the cardiac Tregs than Teffs isolated from infarcted heart, suggesting that CD157 may be a marker for Tregs in injured hearts. However, whether and how the increase of CD157 involved in the function of cardiac Tregs post MI need further exploration. CNT2, a high-affinity adenosine transporter mediating salvage of nucleoside, modulation of purinergic signaling, and energy metabolism in intestinal and liver parenchymal cells, also exhibited a marked elevation in cardiac Tregs at day 7 post-MI 39-41. Borg *et al.* reported no difference in *Cnt2* expression in CD4^+^ T-cells from blood and heart 3 days after I/R injury 23, but our results suggested that CNT2 may also exert its function in injured hearts by regulating purinergic signaling on T cells post-MI. However, there is no difference of *Cnt2* expression between Teffs and Tregs from MI heart.

A2aR is highly expressed in murine T cells 42, and it can be further upregulated by T-cell receptor stimulation and consequently, mediated the inhibition of IFN-γ production 37. Activation of A2aR also inhibits the differentiation of Th1/Th2 cells from naive CD4^+^ T cells 43. We did find increased *A2ar* expression in MCI-H of the MI group compared to sham group, but higher expression of IFN-γ levels in MCI-H in those infarction hearts, suggesting that the elevation of A2aR signaling is insufficient to suppress the IFN-γ content in heart-infiltrating immune cells. In addition, downregulation of *A2aR* expression in Tregs of the MI group may partly explain why Tregs, which infiltrated into the injured heart and expressed high levels of CD73, cannot suppress local inflammation effectively.

In murine AMI models, Tregs are accumulated in the injured heart 7 days after MI and maintained high levels until at least 28 days with a distinct transcriptome 27. Despite their low count in the heart, Tregs, as a subgroup of CD4^+^T cells, are known to regulate the inflammatory and reparative responses post-MI 12, 44, 45. Since we found *Cd73* increased in cardiac Tregs after MI and *Cd73* expression was previously reported to be a key mechanism in maintaining the immunosuppressive function of Tregs 19, 46, we speculated that CD73^+^FoxP3^+^Tregs could migrate to the heart, confer a cardioprotective action, and promote cardiac repair. As expected, administration of CD73KO Tregs, compared to WT-Tregs, showed less efficiency to ameliorate murine heart function and the progress of the LV remodeling at 28 days post-MI. Immune responses mediated by T cells depends on the efficient homing of antigen-primed lymphocytes to antigen-rich nonlymphoid tissues 47, and CD73 regulates T cell migration 48. We further demonstrated that CD73-KO Tregs expressed lower CCR4, C-Met, and CXCR3, which suggested a decreased tissue tropism compared with WT-Tregs 47. Furthermore, our tracking experiments also confirmed this hypothesis by transferring labeled Tregs into MI mice. Also, CD73KO-Tregs displayed lower CD4^+^FoxP3^+^ Tregs in the heart compared to the WT-Tregs group. Since tissue tropism is important for Tregs to exert their function locally 49, 50, the lower number/percentage of Tregs in the injured heart is not sufficient to improve the cardiac healing and the recovery of heart function post MI in mice who received CD73KO Tregs.

Previously, Tregs was confirmed improving healing after MI by modulating different immune cells including Teff 16. In the present study, injection of CD73KO Tregs cannot effectively inhibit the infiltration of CD4^+^ cells into the injured heart. We then investigated the mechanisms that may involve in decreased suppressive effects of CD73KO Tregs compared with WT Tregs. As a crucial transcription factor in the development and function of Tregs 1,27, we found lower level of FoxP3 in our CD73KO Tregs. This is consistent with previous report that loss of CD73 will affect the production of adenosine, which promotes FoxP3 expression in Tregs 30. Decreased FoxP3 affects the formation of repressive chromatin in Tregs upon activation in response to inflammation 51. Furthermore, the nuclear NF-κB subunit p65, which is essential for Treg-dependent maintenance of immune tolerance, decreased significantly in the CD73-deficient Tregs. FoxP3 and the p65 subunit could drive the transcription of CD25 50, and we found CD73KO Tregs exhibited lower intensity and gene level of CD25. This decreases the CD73KO Tregs suppressive function further 52. These differences between WT and CD73KO Tregs may partly uncover the mechanisms of how CD73 affects the function of Tregs.

Expansion of Tregs via the IL2C contributes to heart healing post-MI 13 and attenuates HF progression 34 or cardiac I/R injury 35. In the present study, we demonstrated that the IL2C can significantly increase FoxP3^+^ CD73^+^ Tregs in the murine MI hearts, suggesting another effect of IL2C in MI. Interestingly, IL2C treatment didn't reverse the negative effects on cardiac function in KO mice even though IL2C markedly increased the frequencies of CD4^+^FoxP3^+^Tregs in the spleens and hearts. This highlighted the crucial role of CD73 on Tregs in the MI recovery as well. Mechanically, a previous study revealed that Tregs could modulate the phenotype and function of fibroblast and attenuate the adverse cardiac remodeling post infarction 44. Our result found that supernatant from WT-Tregs but not CD73KO Tregs could ameliorate TGFβ-induced Col1a1 expression in fibroblast. Also, IL2C treatment increased CD73^+^Tregs infiltration in the hearts while decreased the Col1a1 protein level in the injured heart. Together, we consider that the increased CD73^+^Tregs expanded by IL2C can suppress Teffs mediated inflammation, modulate the function of fibroblasts, and subsequently contribute to the recovery of cardiac healing and cardiac function.

Two recent clinical studies revealed a reduced frequency of CD73^+^ lymphocytes in PBMCs of patients with ANCA-associated vasculitis 53, and the involvement of CD73 expressing Tregs in the pathogenesis of human psoriasis 54. We found CD73 expression on CD4^+^ T cells and Tregs was profoundly reduced in PBMCs of AMI patients compared to non-MI patients, which is consistent with our murine MI model. Of note, in the mouse heart both parameters were found to be increased, and in the MI-patient may also have the same enriched phenotype. However, the enriched CD73^+^Tregs in the heart may be not enough to contribute the cardiac healing, that is why expansion of Tregs acts as a new promising strategy to treat ischemic heart disease in a clinical trial (NCT03113773). We further demonstrated a significant negative correlation between CD73 expression on CD4^+^ T cells and serum levels of NT pro-BNP, troponin, myoglobin, creatine kinase isoenzyme and the value of EF. Moreover, logistic regression found that people with high levels of CD73^+^Tregs had much lower MI rate than those with low CD73^+^Tregs levels. This suggests that the peripheral levels of CD73^+^CD4^+^ cells and CD73^+^FoxP3^+^ cells may indirectly reveal the cardiac healing and injury conditions. Moreover, the present clinical and animal results indicate that CD73 on Tregs may participate in cardiac injury and healing after AMI in patents. Further clinical studies are needed to explore whether CD73^+^Tregs can be a prognostic parameter in patients.

## Conclusion

Collectively, the present study initially demonstrated Tregs, accumulating in the infarcted heart, underwent purinergic signaling reprogram with dramatic increase of CD73 expression. Tregs deficient of CD73 showed decreased protective functions in myocardial remodeling induced by MI. The dysfunction of CD73-deficient Tregs may be attributed to the decreased nuclear level of FoxP3. Also, CD73 derived from FoxP3^+^Tregs could bind to CD4^+^FoxP3^-^T cells and inhibit the production of multiple pro-inflammatory cytokines. In addition, administration of low-dose of IL-2/anti-IL-2 complex expanded FoxP3^+^CD73^+^ Tregs and contributed to the recovery of cardiac function. Importantly, we first revealed the negative correlations between circulating percentage of CD73^+^CD4^+^ T cells and NT pro-BNP, troponin, and myoglobin levels in patients with AMI. Furthermore, decreased expression of CD73 in Tregs is associated with AMI patients compared to non-MI patients. Therefore, targeting CD73 on Tregs presents a potential strategy for the treatment of ischemic heart disease.

## Supplementary Material

Supplementary methods, figures and tables.

## Abbreviations

ADP: adenosine diphosphate
AMI: acute myocardial infarction
AMP: adenosine monophosphate
AMP-CP: adenosine 5'-(α, b-methylene) diphosphate
ATP: adenosine triphosphate
CD39: ectonucleoside triphosphate diphosphohydrolase-1
CD73: ecto-5′-nucleotidase
CD73KO: CD73 knockout
CKI: creatine kinase isoenzyme
EF: ejection fraction
FoxP3: Forkhead box P3
FS: fractional shortening
HF: heart failure
IL2C: interleukin-2 and anti-interleukin-2 antibody complex
I/R: ischemia/reperfusion
LV: left ventricular
LVEDD: LV end-diastolic diameter
LVESD: LV end-systolic diameter
LAD: left anterior descending coronary artery
MCI-H: mononuclear cells isolated from heart
MI: myocardial infarction
MLN: mediastinal lymph nodes
PBMCs: peripheral blood mononuclear cells
TAC: transverse aortic constriction
Teffs: effector T-cells
Tregs: regulatory T cells
WT: wild-type
