# Supplementary material for: CD4+FoxP3+CD73+ regulatory T cell promotes cardiac healing post-myocardial infarction
Source: Theranostics. 2022 Mar 6;12(6):2707–21. doi: 10.7150/thno.68437 (PMC8965484; doi:10.7150/thno.68437)
Supplement: Supplementary file 1 — Supplementary methods, figures and tables. [file thnov12p2707s1.pdf]

SUPPLEMENTAL MATERIALS

CD4$^+$FoxP3$^+$CD73$^+$ regulatory T cell promotes cardiac healing post-myocardial infarction

Running title: Zhuang et al.; CD73$^+$Treg promotes cardiac healing

EXPANDED METHODS

Mice

All animals were housed at the animal facility of the Tongji University Animal Center with free access to food and water. All procedures performed in this study were approved by the Tongji University Institutional Animal Care and Use Committee (No. TJLAC-017-025). Timelines of the experiments were described in **Figure 2F**, **3A**, **6D** and **S5A**. At the end of *in vivo* experiments, the mice were euthanized with CO_2 gas.

Human

Blood samples were collected from patients who underwent acute myocardial infarction (AMI) (n=36) up to 7 days after the hospitalization. Age-matched non- myocardial infarction (MI) patients (n=24) with chest pain according to their clinical diagnosis from the Department of Cardiology of Shanghai East Hospital were used as control. Major exclusion criteria were old myocardial infarction, clinically significant other organic heart diseases (such as valvular disease), clinical instability, diseases or medication affecting inflammation, contraindications to tocilizumab, accompanied by other diseases, such as tumors, kidney diseases, autoimmune diseases, etc., suffering from mental illness and unable to achieve informed consent, and any condition that could interfere with protocol adherence. The baseline characteristics of all subjects, shown in **Table S1**, was provided by the clinical laboratory and the Department of Cardiology, Shanghai East hospital, Tongji university School of Medicine. All samples were collected with informed consent from the subjects or their guardians. This study complied with the Declaration of Helsinki and was approved by the Institutional Ethics Committee of Shanghai East Hospital, Tongji University School of Medicine (No. ECSEH2019-004). PBMC were isolated from peripheral blood using Ficoll-Paque PLUS (45-001-750, GE Healthcare) as previously reported [1]. Cells were counted and filtered for the next step.

Murine myocardial infarct model and IL-2/anti-IL-2 complex treatment

To induce the model of MI, mice underwent left anterior descending coronary artery (LAD) ligation as previously reported [2]. In brief, mice were anaesthetized with pentobarbital sodium (50 mg/kg, intraperitoneal injection) for one time, and mechanically ventilated (isoflurane 1–2% vol/vol) with an Inspira - Advanced Safety Ventilator (Harvard Apparatus). After gently opening the skin, fat, muscle, and exposing chest in the 4th intercostal space, the MI group was induced by ligating the LAD branch of the coronary artery permanently with 10.0-prolene suture. In the sham group, a thoracotomy was performed to expose the heart and

the suture was placed but not ligated. The chest was then closed in layers using 5.0 silk and the mice were then weaned off pentobarbital sodium anesthesia.

To evaluate the effect of IL-2/anti-IL-2 complex (IL2C) on amplification of CD73^{+}Treg cells and the therapeutic effect, mice were allocated into the IL2C- or PBS- treated groups randomly in both WT mice and CD73 KO mice. IL2C contained 1.5μg IL-2(Cat. #212-12, Pepro Tech, Rocky Hill, NJ) and 7.5μg anti-IL-2 mAb (JES6-1, Cat:554424, BD Pharmingen, San Jose, CA) as mentioned before [3, 4]. In the IL2C group, complex was incubated at 37°C for 30 min, and then administered intraperitoneally to mouse for seven consecutive days. PBS were used as control. After pre-treatment with IL-2-mAb complex for seven days, mice were subjected to LAD ligation. The timeline of the experiments was described in **Figure S5A, 6D**.

Echocardiography

On 0-, 7- and 28-day after operation, cardiac function of mice was assessed by using the Visual Sonics high-resolution Vevo2100 ultrasound system (VisualSonics Inc., Canada) with a 30-MHz linear array ultrasound transducer (MS-400, VisualSonics Inc., Canada) as previously described [5, 6]. Briefly, mice were anesthetized with light (~1%) isoflurane until the heart rate stabilized at 400 to 500 beats per minute. Parasternal long-axis images were acquired in B-mode with appropriate position of the scan head to identify the maximum LV length. In this view, the M-mode cursor was positioned perpendicular to the maximum LV dimension in end-diastole and systole, and M-mode images were obtained for measuring wall thickness and chamber dimensions. LV ejection fraction and fractional shortening were calculated automatically. The analysis was performed blinded to mice identity.

Histological analysis

Tissues were fixed in 4% paraformaldehyde (PFA), embedded in paraffin and sectioned at 6 μm interval, and cryostat at 8 μm interval. Serial sections were stained with Hematoxylin and eosin (H&E), Masson's trichrome for detection of cardiac fibrosis and Alexa FluorTM 488 conjugated wheat germ agglutinin (WGA) (W11261, Invitrogen) for measurement of cardiomyocyte size in vivo by myocyte cross-section areas according to previous methods [6, 7]. Antibodies including anti-CD3 (ab56313, Abcam, Inc. Cambridge, UK), anti-CD4 (ab183685, Abcam, Inc., Cambridge, UK), anti-FoxP3 (12635, Cell Signaling Technology, Inc. Danvers, MA), and anti-Collagen I (NBP1-30054, Novus Biologicals, Littleton, CO) were used for immunohistochemistry or immunofluorescence (IF) staining. The sections were observed and photographed with microscope (Leica DM6000B, Leica Microsystems, Germany). The percentage of positive cells was quantified by using Image-Pro Plus 6.0 software (Media

Cybernetics, Inc., Rockville, MD, USA). To quantify the percentage of $CD3^{+}CD4^{+}$cells, and $CD4^{+}FoxP3^{+}$ cells, 5 fields were randomly selected from each peri-infarct area in cardiac sections and calculated by the number of double positive cells.

Mononuclear cell preparation for flow cytometry

Mononuclear cells for flow cytometry were isolated from the spleens, mediastinal lymph nodes (MLN) and hearts. Cells from spleens and MLN were isolated by grinding and filtering through 70μm strainer. Single cells from heart tissue were acquired similarly as previously described [8]. In brief, hearts were perfused with pre-cold 1X PBS and cut transversely into two halves. The further mechanically dissociation was performed in the gentleMACS C tubes placed on the dissociator (Miltenyi Biotec, USA). The digestion was continued in 5ml HBSS buffer contained Collagenase II (Worthington, 1.5mg/ml), Collagenase IV (Worthington, 1.5mg/ml) and DNase I (Sigma, 60U/ml). Heart tissues in digestion solution were incubated at 37℃ for 30min at a speed of 200 rpm. After secondly mechanical separation on dissociator, debris in the samples were depleted by the Debris Removal Solution (130-109-398, Miltenyi Biotec, Germany). And then samples were resuspended to obtain single cell suspensions for next step.

Flow cytometry

After preparing the single cell suspension according to the above methods, the samples from murine spleen, blood, MLN, heart and human PBMCs, sequentially filtered through a 40-μm nylon mesh. Followed the manufacturer's instructions, added appropriately fluorescently labeled antibodies at predetermined optimum concentrations and incubated on ice for 20 minutes in the dark for cell-surface staining. After washing with PBS, centrifuging at 350xg for 5 minutes, samples were resuspended for flow cytometric analysis (BD FACSVerse, or BD FACSAria II, BD Biosciences, San Jose, CA). For FoxP3 [9] or T-bet [10] intracellular staining, 1 ml of 1X BioLegend's FoxP3 Fix/Perm solution (Cat.421403, BioLegend, San Diego, CA) or True-Nuclear Transcription Factor Buffer Set (Cat.424401, BioLegend, San Diego, CA) were added to each tube, then vortexed and incubated at room temperature in the dark for 20 minutes. After washing, resuspended cells in 1ml 1X BioLegend's FoxP3 Perm buffer (Cat.421403, BioLegend, San Diego, CA) and incubated at room temperature in the dark for 15 minutes. Add appropriate amount of fluorochrome conjugated anti-FoxP3 antibody or anti-T-bet antibody and incubated at room temperature in the dark for 30 minutes. After washing twice with PBS, centrifuging at 350xg for 5 minutes, samples were resuspended for flow cytometric analysis.

The antibodies for flow cytometry were attached in Table S2. Isotype controls were used in all cases.

Imaging flow cytometry

After sorting, the $CD4^+CD25^+$ T cells from WT or CD73-/- mice were stained with anti-CD4-PE/cy7, anti-FoxP3-AF647 and DPAI as above mentioned. As described in the previous paper [11], cell images were acquired in ImageStream^X MK II (Amnis, Luminex Corporation, USA) Imaging Flow Cytometer and analyzed using IDEAS 6.2 software (Amnis, Luminex Corporation, USA). Nuclear colocalization wizards available in the software guided the analyses. Parameters were evaluated in $CD4^+CD25^+FoxP3^+$ cells (1000-5,000 cells per group). The coefficient of similarity (Cs) was defined as “Bright field similarity” in channels corresponding to FoxP3 and DAPI, or p65 and DAPI.

Cell purification

$CD4^+$ T cell: $CD4^+$ T cells from spleens, MLN and heart were purified with the Dynabeads Untouched Mouse CD4 Cells Kit (Invitrogen, 11415D) [12].

$CD4^+CD25^+$ T cell: Purification of $CD4^+CD25^+$ Tregs from spleens of WT or CD73-/- mice were performed by using Dynabeads Mouse $CD4^+$ $CD25^+$ Regulatory T Cell Isolation Kit (130-091-041, Miltenyi Biotec, Germany) [13].

$CD3^+CD4^+FoxP3^+$ and $CD3^+CD4^+FoxP3^-$ cell: C57BL/6 Foxp3-YFP knock-in mice were adopted to separate $CD3^+CD4^+FoxP3^{YFP+}$ and $CD3^+CD4^+FoxP3^{YFP-}$cells from spleen and heart tissues by flow cytometry (BD FACSAria II Special Order System, BD Biosciences, San Jose, CA).

Cell infusion

On 1-day post-MI, purified $CD4^+$ or $CD4^+CD25^+$ T cells (2×10^5/ 100ul per mouse) were injected in the tail vein for tracing. Cells were stained with the DiR loading solution (Invitrogen, D12731) before adoptive transplantation to observe the distribution in heart by the Small Animal Imaging System (Pearl® Trilogy, LI-COR Biosciences, USA) (Figure 2D, 4B) after being perfused.

To illustrate the therapeutic effect of $CD73^+$ Tregs on MI, $CD4^+CD25^+$ T cells (1×10^6/ 100ul per mouse) from WT or CD73-/- mice were respectively injected in the tail vein of WT mice on 1-dpo. After 7 days and 28 days, Echo was performed for evaluating the cardiac function. And the tissue samples were collected to analyze further.

To confirm the source of cardiac Treg, $CD3^+CD4^+FoxP3^{YFP}$Tregs was isolated from FoxP3-YFP knock-in mice (sham mice) and transferred to MI mice by tail vein injection.

Exosome isolation

Exosomes were isolated from WT and CD73KO Tregs supernatants based on our previous work [14] and followed a previous paper [15]. In brief, splenic WT and CD73KO Tregs was isolated as mentioned above, then cells were cultured overnight in exosome-free media in the present of ™ mouse T-Activator CD3/CD28 dynabeads (11452D, Gibco, Thermo Fisher). Then the culture media was collected, and the supernatant was centrifuged at 2000 × g for 10 min to remove the debris, and then 10000 × g for 10 min at 4°C. Then the supernatant was centrifuged at 120,000 g for 2 h at 4°C to pellet all exosomes (Optima L-100XP Ultracentrifuge, Beckman Coulter). After one wash with PBS, the exosomes were obtained and resuspended in 50 µL PBS.

Cell culture

Tregs ($CD4^{+}CD25^{+}$) were isolated from spleen of WT or CD73KO mice as mentioned above. And Teffs ($CD4^{+}CD25^{-}$) also were purified from WT mice spleen. Each of $CD4^{+}CD25^{+}$ Tregs were co-cultured with Teff cells in the present of CD3/CD28 beads by using the transwell with 1um aperture. After 3d co-cultivation, the Teff cells were obtained and stain with anti-Ki67 for suppression assay in vitro.

$CD3^{+}CD4^{+}FoxP3^{-YFP+}$ Tregs were sorted from FoxP3-YFP murine spleen as mentioned above, labeled with CD73-PE antibody, and washed. Then the labeled cells were placed into the co-culture system with unlabeled Teffs. For Blockade of exosome generation, GW4869(10uM, D1692, Sigma- Aldrich), a neutral sphingomyelinase inhibitor, was used in the coculture also. After culturing for 12h, cells were respectively collected for flow analysis, mRNA isolation, or stained with DAPI for CD73 translocation by confocal microscope (Leica TCS SP8 STED 3X, Leica Microsystems, Germany).

After purifying the Teffs from spleen, the isolated exosomes from WT/ CD73KO mice were used for treating the Teffs in the present of ™ mouse T-Activator CD3/CD28 dynabeads (11452D, Gibco, Thermo Fisher). Then the supernatants were collected for ELISA.

Cardiac fibroblasts were cultured in Dulbecco's Modified Eagle Medium (DMEM) with 10% fetal bovine serum, 1% penicillin (100 U/ml) and 1% streptomycin (100 µg/ml) and were incubated at 37°C in a humidified atmosphere of 95% air and 5% CO2. Then the cells were treated with TGFβ for 24 h with/without WT/KO-Treg supernatant, and harvested at 24 hours for further experiments.

Gene expression

After euthanasia, the hearts were collected and divided into peri-infarct and infarct area and remote area for mouse gene expression microarray (Cat. 026655, Agilent, Santa Clara, CA).

Total mRNA was isolated from the heart peri-infarct area using Trizol (Invitrogen; Thermo Fisher Scientific, Inc., USA) according to the manufacturer's instructions and cDNA was synthesized using a Prime Script RT reagent kit (TaKaRa, Japan).

Sorted FoxP3$^+$ Tregs and FoxP3$^-$ cells were sorted from the heart from 3-4 pooled MI mice and 5-6 pooled sham mice. Then, the sorted cells were pre-amplificated by using QIAseq FX Single Cell RNA Library Kit (180733, Qiagen, Germany). Quantitative real-time PCR was performed as described before [16]. Primer pairs are available in the **Table S3**.

Protein expression

Murine heart tissues were collected, and the protein concentrations were quantified using a bicinchoninic acid (BCA) protein assay kit (Thermo Fisher, USA). After incubating with the following primary antibodies: β-actin (8457, Cell Signaling Technology, USA), anti-CD73(13160, Cell Signaling Technology, USA), anti-Collagen I (NBP1-30054, Novus Biologicals, Littleton, CO) , and anti-Collagen III (ab7778, Abcam, Cambridge, UK). The bands were visualized using an enhanced chemiluminescence (ECL) system. The intensity of each protein band was quantified using Quantity One software (Bio-Rad Laboratories, CA, USA).

For cytokine protein expression, the samples of peri-infarct area in the heart (**Figure S1D**) were collected for immunoassay (Cat. EPX110-20820-901, EPX01A-20614-901, EPX01A-26001-901, EPX01A-26005-901 and EPX01A-26009-901, Thermo Fisher, USA), and the supernatant from Teffs culture system were collected for ELISA.

Statistical analysis

All data are presented as mean ± standard error of the mean (SEM). All data were checked for normality and equal variance before analysis by Shapiro-Wilk test. Data are analyzed by SPSS 11.0 (SPSS Inc., USA) statistical software and GraphPad Prism 8 statistical software (GraphPad Software Inc, San Diego, California). Comparisons between two groups were analyzed by unpaired Student’s t-test. One-way ANOVA with Tukey post hoc tests was used for comparisons between multiple groups; and two-way ANOVA was used for comparisons between multiple groups when there were 2 experimental factors. For comparison of composition ratios in clinical data, Pearson's chi-squared test or, if not suitable, Yates' corrected chi-squared test was performed. Spearman’s rank correlation was used to assess

the relationship between the level of NT-pro BNP, troponin, myoglobin, CKI, and the proportion of $CD4^{+}CD73^{+}$ cells in PBMCs in patients. Logistic regression model was set up to show the relationship between the percentage of CD73 in $CD4^{+}$T cells and MI, and the percentage of CD73 in Tregs and MI. Models also adjusted by age, gender, BMI, systolic blood pressure value, diastolic blood pressure value, total cholesterol, triglyceride, low density lipoprotein, high density lipoprotein and fasting blood glucose. *P* value of <0.05 was considered as statistical significance.

SUPPLEMENTARY TABLE

Table S1. Baseline

	**MI patients** n = 36	**Non-MI patients** n = 24	χ^2	***P*-value**
**Demographics**				
Age, Mean (SD), years	66.22 (11.66)	66.04 (6.53)		0.9455
Female, n (%)	18 (50.00%)	15 (62.50%)	0.9091	0.3404
**Clinical signs**				
BMI, Mean (SD), kg/m2	24.19 (2.90)	25.46 (2.50)		0.0848
HR, Mean (SD), /min	84.19 (18.39)	83.46 (15.12)		0.8724
SBP, Mean (SD), mmHg	126.33 (30.79)	130.42 (16.90)		0.5556
DBP, Mean (SD), mmHg	72.36 (16.43)	78.50 (8.05)		0.0951
**Medical history**				
Diabetes, n (%)	15 (41.67%)	5 (20.83%)	2.813	0.0935
Hypertension, n (%)	20 (55.56%)	13 (54.17%)	0.01122	0.9156
**Cardiac function**				
NYHA Degree I, n (%)		17		
Degree II, n (%)		7		
Degree III, n (%)		0		
Degree IV, n (%)		0		
Killip Degree I, n (%)	26			
Degree II, n (%)	6	/		
Degree III, n (%)	0	/		
Degree IV, n (%)	4	/		
**Echocardiograph**				
EF Mean (SD), %	51.97 (9.46)	64.20 (5.32)		**<0.0001******
FS Mean (SD), %	33.43 (2.44)	35.38 (3.55)		**0.0143***
**Coronary angiography**				
LM stenosis, n (%)	8 (22.22%)	0 (0)	4.381	**0.0363***
LAD stenosis, n (%)	32 (88.89%)	8 (33.33%)	20	**<0.0001******
LCX stenosis, n (%)	31 (86.11%)	3 (12.50%)	31.78	**<0.0001******
RCA stenosis, n (%)	30 (83.33%)	7 (29.17%)	17.87	**<0.0001******
**Medication**				

ACEI or ARB, n (%)	17 (47.22%)	10 (41.67%)	0.1796	0.6717
Beta-blocker, n (%)	24 (66.67%)	14 (58.33%)	0.4306	0.6562
MRA, n(%)	15 (41.67%)	1 (4.17%)	10.36	0.0013**
LD, n (%)	14 (38.89%)	1 (4.17%)	9.259	0.0023**
Nitrate, n (%)	8 (22.22%)	0 (0)	4.381	0.0363*
Statins, n (%)	36 (100%)	19 (79.17%)	5.682	0.0171*
**Laboratory measurements**				
NT-pro BNP, Mean (SD), ng/L	2035.30 (2651.81)	81.57 (134.78)		0.0007***
CTnI, Mean (SD), ng/mL	4.03 (3.59)	0.01 (0.01)		<0.0001****
Myo, Mean (SD), ng/mL	749.72 (978.49)	26.67 (14.89)		0.0006***
CKI, Mean (SD), ng/mL	111.30 (93.97)	1.74 (1.78)		<0.0001****
Scr, Mean (SD), umol/L	81.14 (26.22)	65.80 (11.09)		0.0090**
BUN, Mean (SD), mmol/L	24.04 (77.27)	5.96 (1.54)		0.2578
BUA, Mean (SD), umol/L	335.81 (117.42)	333.46 (81.67)		0.9324
e-GFR, Mean (SD), ml/min	111.83 (25.47)	119.20 (37.72)		0.3694
TC, Mean (SD), mmol/L	3.83 (1.66)	3.85 (2.17)		0.9679
TG, Mean (SD), mmol/L	2.43 (1.27)	2.16 (1.16)		0.4073
LDL, Mean (SD), mmol/L	2.73 (0.97)	2.53 (1.03)		0.4483
HDL, Mean (SD), mmol/L	1.28 (0.66)	1.24 (0.32)		0.7882
ALT, Mean (SD), U/L	129.54 (147.96)	23.60 (16.46)		0.0009**
AST, Mean (SD), U/L	119.68 (217.87)	19.93 (8.55)		0.0293*
FBG, Mean (SD), mmol/L	7.03 (2.46)	6.04 (1.37)		0.0784

HR, heart rate; SBP, systolic blood pressure; DBP, diastolic blood pressure; EF, ejection fraction; FS, shortening fraction; LM, left main coronary artery; LAD, left anterior descending branch; LCX, left circumflex branch; RCA, right coronary artery; ACEI, angiotensin converting enzyme inhibitor; ARB, angiotensin receptor blocker; MRA, mineralocorticoid receptor antagonist; LD, loop diuretic; Pro-BNP, pro-brain natriuretic peptide; CTnI, cardiac troponin I; Myo, myoglobin; CKI, creatine kinase isoenzymes; Scr, Serum creatinine; BUN, blood urea nitrogen; BUA, blood uric acid; e-GFR, estimated glomerular filtration rate; TC, total cholesterol; TG, triglyceride; LDL, low density lipoprotein; HDL, high density lipoprotein; ALT, alanine aminotransferase; AST, aspartate aminotransferase; FBG, fasting blood glucose. *P<0.05, **P<0.01, ***P<0.001, **** P<0.0001. For comparison of composition ratios in clinical data, Pearson's chi-squared test or, if not suitable, Yates' corrected chi-squared test was performed; others, unpaired Student’s t-test.

Table S2. Overview of fluorescence labeled antibodies used for fluorescence associated cell analysis and sorting, indicating the name, fluorochrome, catalog and trade name

Name	Fluorochrome	Catalog	Trade name
anti-human CD3	PE-Cyanine7	25-0038-42	eBioscience
anti-human CD4	PerCP-Cy5.5	317428	BioLegend
anti-human CD127	PE	12-1278-42	eBioscience
anti-human CD25	APC	356110	BioLegend
anti-human CD25	BV421	562443	BD Horizon
anti-human CD73	APC	344006	BioLegend
anti-human CD73	PE-Cyanine7	344022	BioLegend
anti-human FoxP3	Alexa Fluor 488	53-4776-42	eBioscience
anti-human/mouse FoxP3	Alexa Fluor 647	320008	BioLegend
anti-mouse CD3	PE	100308	BioLegend
anti-mouse CD3	PE-Cyanine7	100220	BioLegend
anti-mouse CD4	PerCP-Cy5.5	100434	BioLegend
anti-mouse CD25	BV421	562606	BD Horizon
anti-mouse CD73	APC	127210	BioLegend
anti-mouse CD73	PE-Cyanine7	127224	BioLegend
anti-mouse CD45	APC	103112	BioLegend
anti-mouse CD45	FITC	103108	BioLegend
anti-mouse Foxp3	Alexa Fluor 488	53-5773-82	eBioscience
anti-mouse p65	APC	653005	BioLegend
anti-mouse T-bet	BV421	563318	BD Horizon
anti-mouse CCR4	APC	131211	BioLegend
anti-mouse C-Met	FITC	11-8854-80	Invitrogen
anti-mouse CXCR3	PerCP-Cy5.5	126513	BioLegend
anti-mouse TGF-β	PE	141404	BioLegend
anti-mouse IL-10	FITC	505006	BioLegend
anti-mouse Helios	PE-Cyanine7	137235	BioLegend
anti-mouse CD103	PE	121406	BioLegend

Table S3. Overview of Primer pairs used in the study.

Gene		5‘-3’
Cxcl10	Forward Primer	CCAAGTGCTGCCGTCATTTTC
	Reverse Primer	GGCTCGCAGGGATGATTTCAA
Ccl8	Forward Primer	CTGGGCCAGATAAGGCTCC
	Reverse Primer	CATGGGGCACTGGATATTGTT
Ccl3	Forward Primer	TTCTCTGTACCATGACACTCTGC
	Reverse Primer	CGTGGAATCTTCCGGCTGTAG
Ccr2	Forward Primer	ATCCACGGCATACTATCAACATC
	Reverse Primer	CAAGGCTCACCATCATCGTAG
Ccr1	Forward Primer	CTCATGCAGCATAGGAGGCTT
	Reverse Primer	ACATGGCATCACCAAAAATCCA
Cxcl5	Forward Primer	TCCAGCTCGCCATTCATGC
	Reverse Primer	TTGCGGCTATGACTGAGGAAG
Ccl7	Forward Primer	GCTGCTTTCAGCATCCAAGTG
	Reverse Primer	CCAGGGACACCGACTACTG
Ccl2	Forward Primer	CCAACCACCAGGCTACAGG
	Reverse Primer	GCGTCACACTCAAGCTCTG
Ccr5	Forward Primer	TTTTCAAGGGTCAGTTCCGAC
	Reverse Primer	GGAAGACCATCATGTTACCCAC
Ccl12	Forward Primer	ATTTCCACACTTCTATGCCTCCT
	Reverse Primer	ATCCAGTATGGTCCTGAAGATCA
Cxcr4	Forward Primer	GAAGTGGGGTCTGGAGACTAT
	Reverse Primer	TTGCCGACTATGCCAGTCAAG
Ccl9	Forward Primer	CCCTCTCCTTCCTCATTCTTACA
	Reverse Primer	AGTCTTGAAAGCCCATGTGAAA
Cx3cr1	Forward Primer	GAGTATGACGATTCTGCTGAGG
	Reverse Primer	CAGACCGAACGTGAAGACGAG
Ccl6	Forward Primer	GCTGGCCTCATACAAGAAATGG
	Reverse Primer	GCTTAGGCACCTCTGAACTCTC
Cd4	Forward Primer	TCCTAGCTGTCACTCAAGGGA
	Reverse Primer	TCAGAGAACTTCCAGGTGAAGA
Cd73	Forward Primer	ACGTGCTGTTTTTGGATGCC
	Reverse Primer	AGTGCCATAGCATCGTAGCC
Tgf-β	Forward Primer	CTCCCGTGGCTTCTAGTGC
	Reverse Primer	GCCTTAGTTTGGACAGGATCTG
Il-10	Forward Primer	GCTCTTACTGACTGGCATGAG
	Reverse Primer	CGCAGCTCTAGGAGCATGTG
Helios	Forward Primer	GAGCCGTGAGGATGAGATCAG
	Reverse Primer	CTCCCTCGCCTTGAAGGTC
Cd103	Forward Primer	CCTGTGCAGCATGTAAAAGAATG
	Reverse Primer	CAAGGATCGGCAGTTCAGATAC
Panx1	Forward Primer	GAGCGAGTCTGGAAACCTCC
	Reverse Primer	GGGCAGGTACAGGAGTATGG
Cx43	Forward Primer	TGGCCTGCTGAGAACCTACA
	Reverse Primer	CAGAGCGAGAGACACCAAGGA
Cx37	Forward Primer	CCCACATCCGATACTGGGTG
	Reverse Primer	CGAAGACGACCGTCCTCTG
Cd39	Forward Primer	AAGGTGAAGAGATTTTGCTCCAA
	Reverse Primer	TTTGTTCTGGGTCAGTCCCAC

Alpi	Forward Primer	GCAGTGCCTCAGACCCTTAC
	Reverse Primer	ATGAGAGCCCGTTGTAGGTG
Enpp1	Forward Primer	TGAGAGCTGTACGCATGGGA
	Reverse Primer	GGCCAGTGATGAGTTCCACG
Enpp3	Forward Primer	CAGTTGACAATGCCTTTGGAATG
	Reverse Primer	CACTCTATCACAGGAGGTCTGG
Art2b	Forward Primer	AAGGGCTCTGTGCGATTTGG
	Reverse Primer	CTCCTCTTCACGAGGGAATGA
Cd157	Forward Primer	ACTACCAGTCCTGCCCCACAT
	Reverse Primer	AAAAACCCTCTCGTGGGATAGG
Cd38	Forward Primer	TCTCTAGGAAAGCCCAGATCG
	Reverse Primer	GTCCACACCAGGAGTGAGC
Ada	Forward Primer	ACCCGCATTCAACAAACCCA
	Reverse Primer	AGGGCGATGCCTCTCTTCT
Adk	Forward Primer	GGACCGTGATCTTCACACAAG
	Reverse Primer	GCGAATGCACTCAGTCAGAG
Ent1	Forward Primer	CGACTGATGCCCGCTTACTC
	Reverse Primer	GGGAGGGACATCAGGTCACA
Ent2	Forward Primer	TCATTACCGCCATCCCGTACT
	Reverse Primer	CCCAGTTGTTGAAGTTGAAAGTG
Cnt2	Forward Primer	AGTGGAGAATTGCATGGAGAAC
	Reverse Primer	GACCAAGCAGGATCTTTCTGAA
A1	Forward Primer	TGGTGATTTGGGCTGTGAAG
	Reverse Primer	ATCAGCTACCGCCAGGGATA
A2a	Forward Primer	TTCCACTCCGGTACAATGGC
	Reverse Primer	CGATGGCGAATGACAGCAC
A2b	Forward Primer	AGCTAGAGACGCAAGACGC
	Reverse Primer	GTGGGGGTCTGTAATGCACT
A3	Forward Primer	AAGGTGAAATCAGGTGTTGAGC
	Reverse Primer	AGGCAATAATGTTGCACGAGT
Ccr4	Forward Primer	ATCCTGAAGGACTTCAAGCTCCA
	Reverse Primer	AGGTCTGTGCAAGATCGTTTCATGG
C-Met	Forward Primer	TCCTGCACTGTGAGCATTTC
	Reverse Primer	ACGATTGGGTTTCAGCAGAC
Cxcr3	Forward Primer	GTGGCTGCTGTGCTACTGAG
	Reverse Primer	AAGGCCCCTGCATAGAAGTT
Foxp3	Forward Primer	CCCATCCCCAGGAGTCTTG
	Reverse Primer	ACCATGACTAGGGGCACTGTA
Cd25	Forward Primer	TGGTCTATATGCGTTGCTTGCTTAGG
	Reverse Primer	TTCTCGATTTGTCATGGGAGT
Il-1β	Forward Primer	GCAACTGTTCCTGAACTCAACT
	Reverse Primer	ATCTTTTGGGGTCCGTCAACT
Tnf-α	Forward Primer	CCTGTAGCCCACGTCGTAG
	Reverse Primer	GGGAGTAGACAAGGTACAACCC
Ifn-γ	Forward Primer	ACAGCAAGGCGAAAAAGGATG
	Reverse Primer	TGGTGGACCACTCGGATGA
Il-17	Forward Primer	TTTAACTCCCTTGGCGCAAAA
	Reverse Primer	CTTTCCCTCCGCATTGACAC
Mmp2	Forward Primer	CAAGTTCCCCGGCGATGTC
	Reverse Primer	TTCTGGTCAAGGTCACCTGTC
α -Sma	Forward Primer	GTCCCAGACATCAGGGAGTAA

	Reverse Primer	TCGGATACTTCAGCGTCAGGA
Anp	Forward Primer	GCTTCCAGGCCATATTGGAG
	Reverse Primer	GGGGGCATGACCTCATCTT
Bnp	Forward Primer	AGTCCTTCGGTCTCAAGGCA
	Reverse Primer	CCGATCCGGTCTATCTTGTGC
Bcl2	Forward Primer	GTCGCTACCGTCGTGACTTC
	Reverse Primer	CAGACATGCACCTACCCAGC
Bax	Forward Primer	TGAAGACAGGGGCCTTTTTG
	Reverse Primer	AATTCGCCGGAGACACTCG
β-actin	Forward Primer	GGCTGTATTCCCCTCCATCG
	Reverse Primer	CCAGTTGGTAACAATGCCATGT

Table S4 Risk factors and parameters for the relationship between the percentage of CD73 in $CD4^{+}$T cells and MI in the logistic regression models

	***P* Value**	**OR**	**95% CI**
**-Unadjusted**			
**Level of CD73+/CD4+**			
**≤ 9.57**	0.013	4.000	1.337-11.965
**> 9.57**		1	
**-Adjusted**			
**Level of CD73+/CD4+**			
**≤ 9.57**	0.042	7.663	1.080-54.355
**> 9.57**		1	
**Age**	0.096	0.910	0.813-1.017
**Gender**	0.015	0.075	0.009-0.603
**BMI**	0.096	0.760	0.551-1.050
**SBP**	0.595	1.016	0.959-1.076
**DBP**	0.088	0.898	0.793-1.016
**TC**	0.546	0.793	0.374-1.683
**TG**	0.699	1.212	0.457-3.213
**LDL**	0.129	4.067	0.666-24.854
**HDL**	0.668	1.410	0.294-6.765
**FBG**	0.034	1.725	1.043-2.851

BMI, body mass index, SBP, systolic blood pressure; DBP, diastolic blood pressure, TC, total cholesterol; TG, triglyceride; LDL, low density lipoprotein; HDL, high density lipoprotein; FBG, fasting blood glucose.

Table S5 Risk factors and parameters for the relationship between the percentage of CD73 in Tregs and MI in the logistic regression models

	P Value	OR	95% CI
**-Unadjusted**			
**Level of CD73+/Tregs**			
**≤ 10.07**	0.009	4.333	1.439-13.047
**> 10.07**		1	
**-Adjusted**			
**Level of CD73+/Tregs**			
**≤ 10.07**	0.030	11.043	1.254-97.238
**> 10.07**		1	
**Age**	0.082	0.895	0.791-1.014
**Gender**	0.012	0.026	0.001-0.454
**BMI**	0.168	0.782	0.551-1.110
**SBP**	0.225	1.034	0.979-1.093
**DBP**	0.027	0.873	0.774-0.985
**TC**	0.294	0.660	0.304-1.433
**TG**	0.736	0.836	0.295-2.369
**LDL**	0.044	9.562	1.063-86.004
**HDL**	0.219	2.849	0.537-15.107
**FBG**	0.036	1.823	1.040-3.196

BMI, body mass index, SBP, systolic blood pressure; DBP, diastolic blood pressure, TC, total cholesterol; TG, triglyceride; LDL, low density lipoprotein; HDL, high density lipoprotein; FBG, fasting blood glucose.

SUPPLEMENTARY FIGURE AND FIGURE LEGENDS

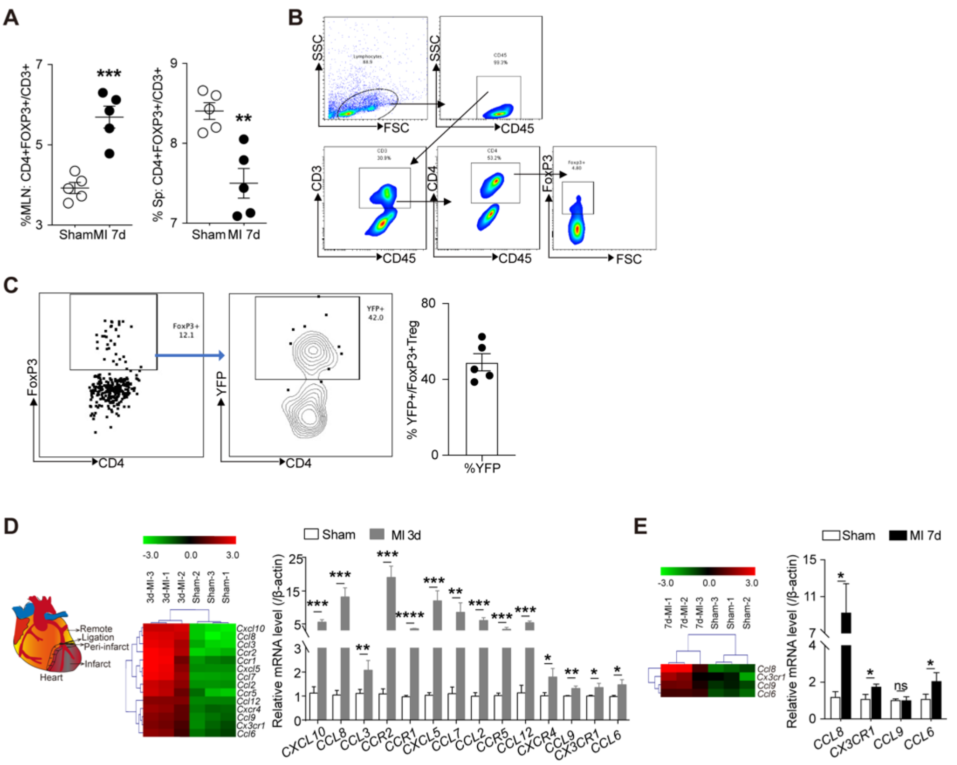

Figure S1 A the percentage of CD4$^+$FoxP3$^+$ cells gated in CD3$^+$ cells in the MLN and spleen. **B,** Analysis strategy of PBMC from mice. **C,** Representative flow cytometry plots and the percentage of YFP$^+$ cell gated on FoxP3$^+$Tregs in the heart after MI. **D-E,** Schematic diagram, clustered heat map of the chemokine and chemokine receptor from peri-infarct area of heart in MI group and apical area in sham group, for 3-day(**D**) and for 7-day(**E**) post-MI, and their PCR validation respectively. MLN, mediastinal lymph nodes; Sp, Spleen; LAD, left anterior descending artery. *P<0.05, **P<0.01, ***P<0.001, **** P<0.0001.

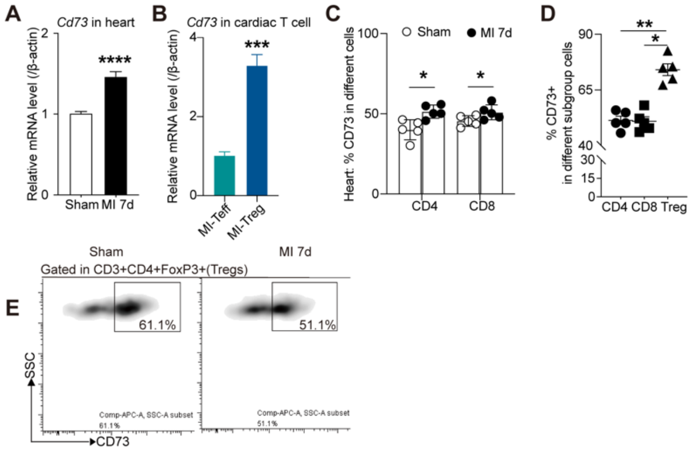

Figure S2 A, mRNA level of *Cd73* in infarct area of heart. **B,** mRNA level of *Cd73* in isolated Teff and Treg cells injured heart. **C,** The percentage of CD73 in $CD4^+$ and $CD8^+$ cells. **D,** The percentage of CD73 in $CD4^+$, $CD8^+$ and $CD4^+FoxP3^+$Treg in the injured heart. **E,** Representative flow cytometry density plots of CD73+ gated in Tregs in PBMC. **P*<0.05, ***P*<0.01, ***P<0.001, **** *P*<0.0001.

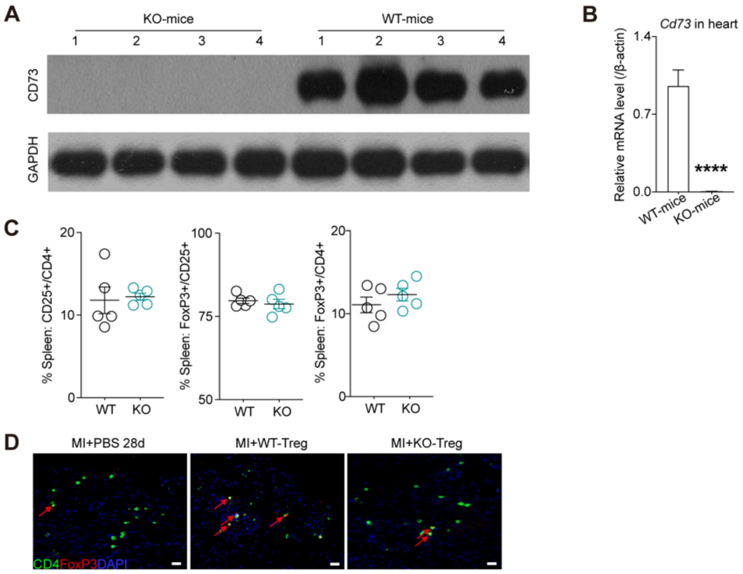

Figure S3 A, Representative western blot of CD73 protein expression in the WT/KO murine heart tissue. **B,** mRNA level of *Cd73* in the heart. **C,** the percentage of CD25+ gated in CD4+, FoxP3+ gated on CD25$^+$ cells, FoxP3+ gated on CD4$^+$ cells. **D,** Representative Immunofluorescence staining of CD4$^+$FoxP3$^+$ T cells, White arrows represent CD4$^+$ cells and red arrows represent CD4$^+$FoxP3$^+$ cells. ****P<0.0001.

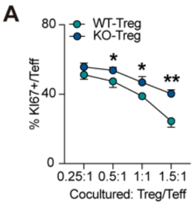

Figure S4 A, the percentage of Ki67+ cells gated in Teff cells after cocultured with Treg cells. **P*<0.05, ***P*<0.01. **A,** Unpaired Student’s t-test.

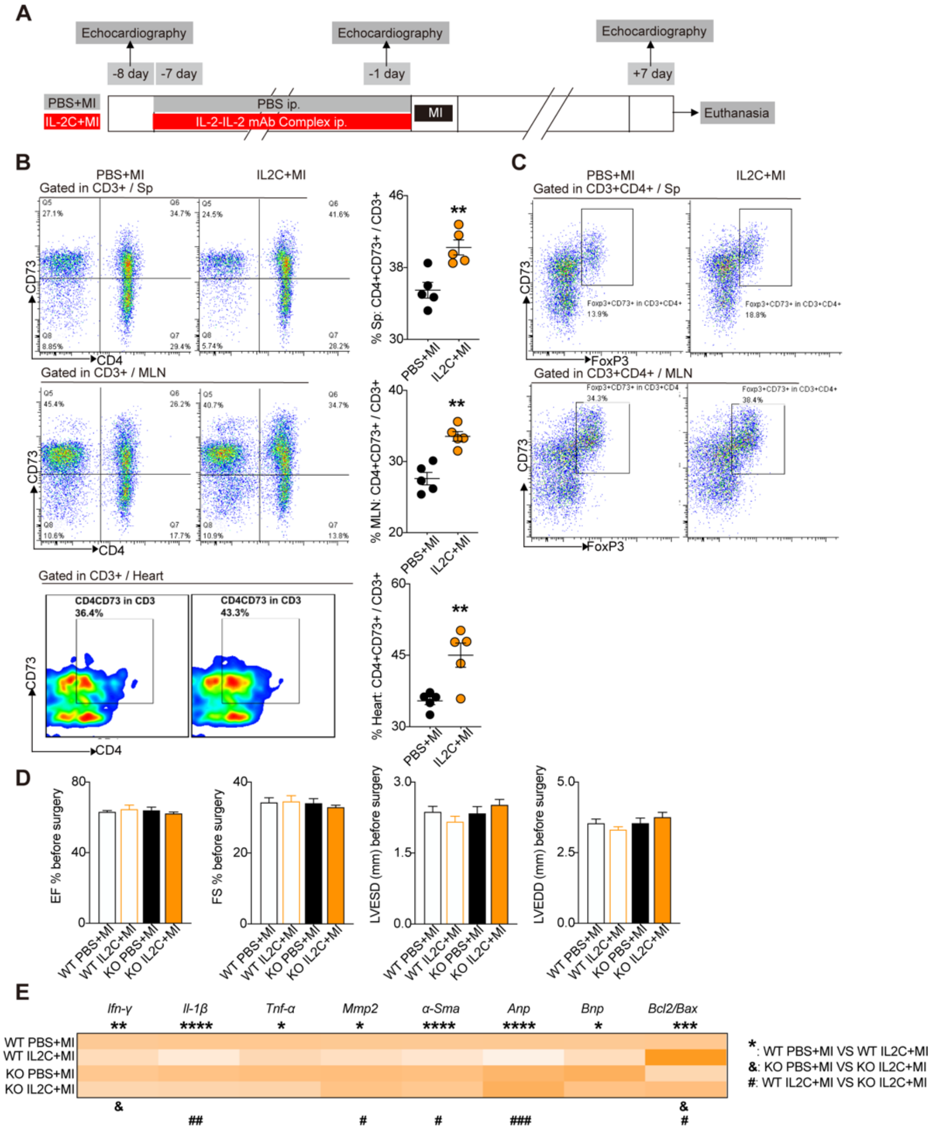

Figure S5 A, Schematic diagram of an in vivo experiment for detect the effect of interleukin-2 and anti-interleukin-2 antibody (IL-2/anti-IL-2) complex injection on CD73$^+$Tregs expansion and its role in the recovery of cardiac function. **B,** Representative flow cytometry plot of CD4$^+$CD73$^+$/CD3+ in spleen, MLN, and heart after IL2C or PBS injection. **C,** Representative flow cytometry pseudocolor of FoxP3$^+$CD73$^+$/CD3$^+$CD4$^+$ in spleen and MLN after IL2C or PBS injection. **D,** Ejection fraction and fractional shortening, and LVESD/LVEDD by echocardiography at day 0. **E,** mRNA levels of inflammatory factors (*Ifn-γ, Il-1β* and *Tnf-α*), myocardial fibrosis markers (*Mmp2* and *α-Sma*), hypertrophy markers (*Anp*, and *Bnp*) and apoptosis marker (*Bcl2/Bax*) in the peri-infarct areas of heart tissues after administration. IL-2C indicates interleukin-2 and anti-interleukin-2 antibody complex; MCI-H indicates mononuclear cells isolated from hearts; MLN, mediastinal lymph nodes; Sp, Spleen. **E**, *: WT PBS+MI group

compared with WT IL2C+MI group; [&]: KO PBS+MI group compared with KO IL2C+MI group; [#]: WT IL2C +MI group compared with KO IL2C+MI group. *$P<0.05$, **$P<0.01$, *** $P<0.001$, **** $P<0.0001$. **B, D,** Unpaired Student's t-test**.**

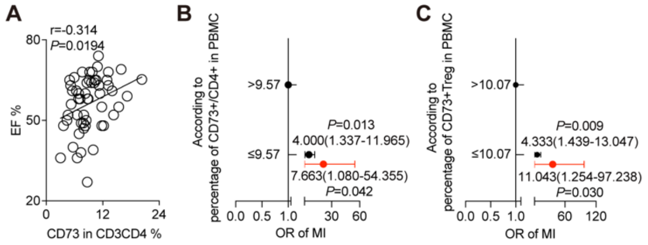

Figure S6 A, Correlation analysis of the EF value and the ratio of CD73+ in $CD3^+CD4^+$ cells in PBMC from AMI and non-MI patients. Line represents linear regression of data. Sites with coefficients, and P values inside plots**. B-C,** Unadjusted (Black) and adjusted (Red) ORs of MI according to the percentage of CD73+ in $CD4^+$ cells (**B**) and the percentage of CD73+ in Tregs (**C**) in PBMCs in those participants with and without AMI by logistic regression. Model adjusted by age, gender, body mass index (BMI), systolic blood pressure value, diastolic blood pressure value, total cholesterol, triglyceride, low density lipoprotein, high density lipoprotein and fasting blood glucose. **A,** Spearman's rank correlation. **B, C,** logistic regression.

SUPPLEMENTARY REFERENCE

1. Corkum CP, Ings DP, Burgess C, Karwowska S, Kroll W, Michalak TI. Immune cell subsets and their gene expression profiles from human PBMC isolated by Vacutainer Cell Preparation Tube (CPT™) and standard density gradient. BMC Immunol. 2015; 16: 48.
2. Hofmann U, Beyersdorf N, Weirather J, Podolskaya A, Bauersachs J, Ertl G, et al. Activation of $CD4^+$ T lymphocytes improves wound healing and survival after experimental myocardial infarction in mice. Circulation. 2012; 125: 1652-63.
3. Boyman O, Kovar M, Rubinstein MP, Surh CD, Sprent J. Selective stimulation of T cell subsets with antibody-cytokine immune complexes. Science. 2006; 311: 1924-7.
4. Webster KE, Walters S, Kohler RE, Mrkvan T, Boyman O, Surh CD, et al. In vivo expansion of T reg cells with IL-2-mAb complexes: induction of resistance to EAE and long-term acceptance of islet allografts without immunosuppression. J Exp Med. 2009; 206: 751-60.
5. Bhan A, Sirker A, Zhang J, Protti A, Catibog N, Driver W, et al. High-frequency speckle tracking echocardiography in the assessment of left ventricular function and remodeling after murine myocardial infarction. Am J Physiol Heart Circ Physiol. 2014; 306: H1371-H83.
6. Liu J, Zhuang T, Pi J, Chen X, Zhang Q, Li Y, et al. Endothelial Forkhead Box Transcription Factor P1 Regulates Pathological Cardiac Remodeling Through Transforming Growth Factor-β1-Endothelin-1 Signal Pathway. Circulation. 2019; 140: 665-80.
7. Zhuang R, Wu J, Lin F, Han L, Liang X, Meng Q, et al. Fasudil preserves lung endothelial function and reduces pulmonary vascular remodeling in a rat model of end-stage pulmonary hypertension with left heart disease. Int J Mol Med. 2018; 42: 1341-52.
8. Li C, Sun X-N, Zeng M-R, Zheng X-J, Zhang Y-Y, Wan Q, et al. Mineralocorticoid Receptor Deficiency in T Cells Attenuates Pressure Overload-Induced Cardiac Hypertrophy and Dysfunction Through Modulating T-Cell Activation. Hypertension. 2017; 70: 137-47.
9. Groh V, Smythe K, Dai Z, Spies T. Fas-ligand-mediated paracrine T cell regulation by the receptor NKG2D in tumor immunity. Nat Immunol. 2006; 7: 755-62.
10. Miyauchi K, Sugimoto-Ishige A, Harada Y, Adachi Y, Usami Y, Kaji T, et al. Protective neutralizing influenza antibody response in the absence of T follicular helper cells. Nat Immunol. 2016; 17: 1447-58.
11. Emmerson A, Trevelin SC, Mongue-Din H, Becker PD, Ortiz C, Smyth LA, et al. Nox2 in regulatory T cells promotes angiotensin II-induced cardiovascular remodeling. J Clin Invest. 2018; 128: 3088-101.
12. Toscano MA, Bianco GA, Ilarregui JM, Croci DO, Correale J, Hernandez JD, et al. Differential glycosylation of TH1, TH2 and TH-17 effector cells selectively regulates susceptibility to cell death. Nat Immunol. 2007; 8: 825-34.
13. Fallarino F, Grohmann U, Hwang KW, Orabona C, Vacca C, Bianchi R, et al. Modulation of tryptophan catabolism by regulatory T cells. Nat Immunol. 2003; 4: 1206-12.
14. Ge X, Meng Q, Wei L, Liu J, Li M, Liang X, et al. Myocardial ischemia-reperfusion induced cardiac extracellular vesicles harbour proinflammatory features and aggravate heart injury. J Extracell Vesicles. 2021; 10: e12072.
15. Smyth LA, Ratnasothy K, Tsang JY, Boardman D, Warley A, Lechler R, et al. CD73 expression on extracellular vesicles derived from $CD4^+$ $CD25^+$ $Foxp3^+$ T cells contributes to their regulatory function. Eur J Immunol. 2013; 43: 2430-40.

16. Wang H, Xu X, Fassett J, Kwak D, Liu X, Hu X, et al. Double-stranded RNA-dependent protein kinase deficiency protects the heart from systolic overload-induced congestive heart failure. Circulation. 2014; 129: 1397-406.
